# DNA i‑Motifs Reversibly Disperse and Modulate the Photophysical Properties of an Aggregated Zinc Phthalocyanine Photosensitizer, Triggered by pH and Temperature

**DOI:** 10.1021/acs.jpclett.6c01427

**Published:** 2026-09-07

**Authors:** Eleanor R. Windle, Nadia Elghobashi-Meinhardt, Robert M. Edkins, Susan J. Quinn

**Affiliations:** † School of Chemistry, 8797 University College Dublin, Dublin 4 D04 V1W8, Ireland; ‡ Department of Pure and Applied Chemistry, 3527 University of Strathclyde, Glasgow G1 1XL, U.K.

## Abstract

Cytosine-rich DNA i-motifs (iM) efficiently and reversibly disperse aggregated cationic zinc phthalocyanine (Pc) photosensitizer **1** into a monomeric DNA-bound form with turned-on near-infrared emission and singlet oxygen (^1^O_2_) photosensitization. Aggregate disruption and binding occur exclusively with folded iMs, enabling pH- and temperature-controlled switching of Pc photophysics. Circular dichroism (CD) titrations and thermal denaturation studies with biologically relevant iMs, including those formed from the human telomere and cMYC oncogene promoter sequences, reveal sequence-dependent dispersion efficiency. Several folded iMs rival G-quadruplex DNA in disrupting Pc aggregates, while unfolded iMs and duplex DNA are ineffective. Spectroscopic and thermal analyses indicate that binding is favored by structural flexibility and partially folded states. Importantly, iM-mediated dispersion switches on reversible ^1^O_2_ photosensitization under acidic conditions. These findings identify DNA iMs as potent and previously unrecognized regulators of Pc photosensitizer aggregation, providing a potential DNA-gated responsive strategy for modulating their photodynamic versus photothermal activity.

## Graphical abstract

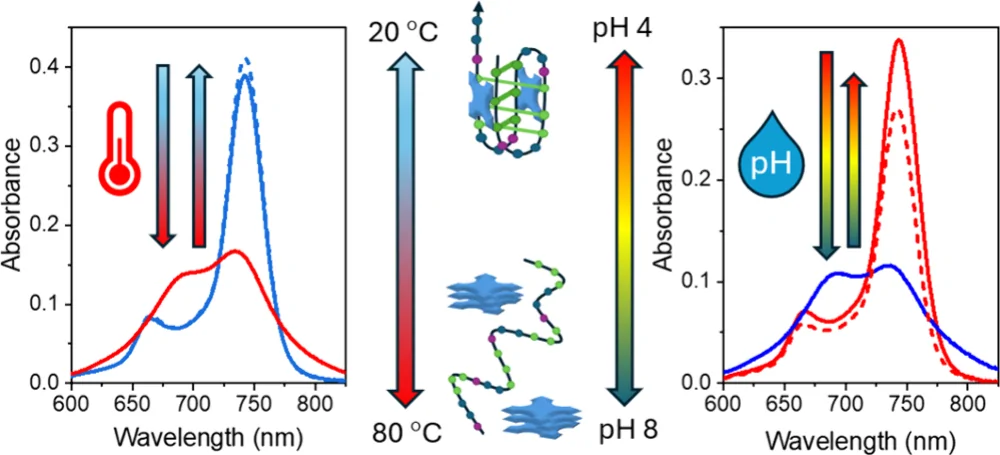

The i-motif (iM) is a unique DNA structure comprising interdigitated base pairs formed by hemiprotonated cytosine-rich nucleic acids (Figure 1a).^1,2^ Experimental studies support the transient formation of iMs in cells and understanding of its biological role continues to evolve.^3–5^ While typically formed at acidic pH, some sequences form iMs at neutral and physiological pH.^6^ Several cytosine-rich DNA iM-forming sequences are complements to guanine-rich quadruplex (G4)-forming sequences, including the human telomere (**G-HT**) and **cMYC** oncogene promoter. There is significant interest in the development of molecular probes capable of recognizing iMs through loop binding, external groove interactions and intercalation.^7^ Examples include small organic molecules,^8,9^ Ru­(II) polypyridyl complexes,^10,11^ and porphyrins.^12,13^

**Figure 1:**
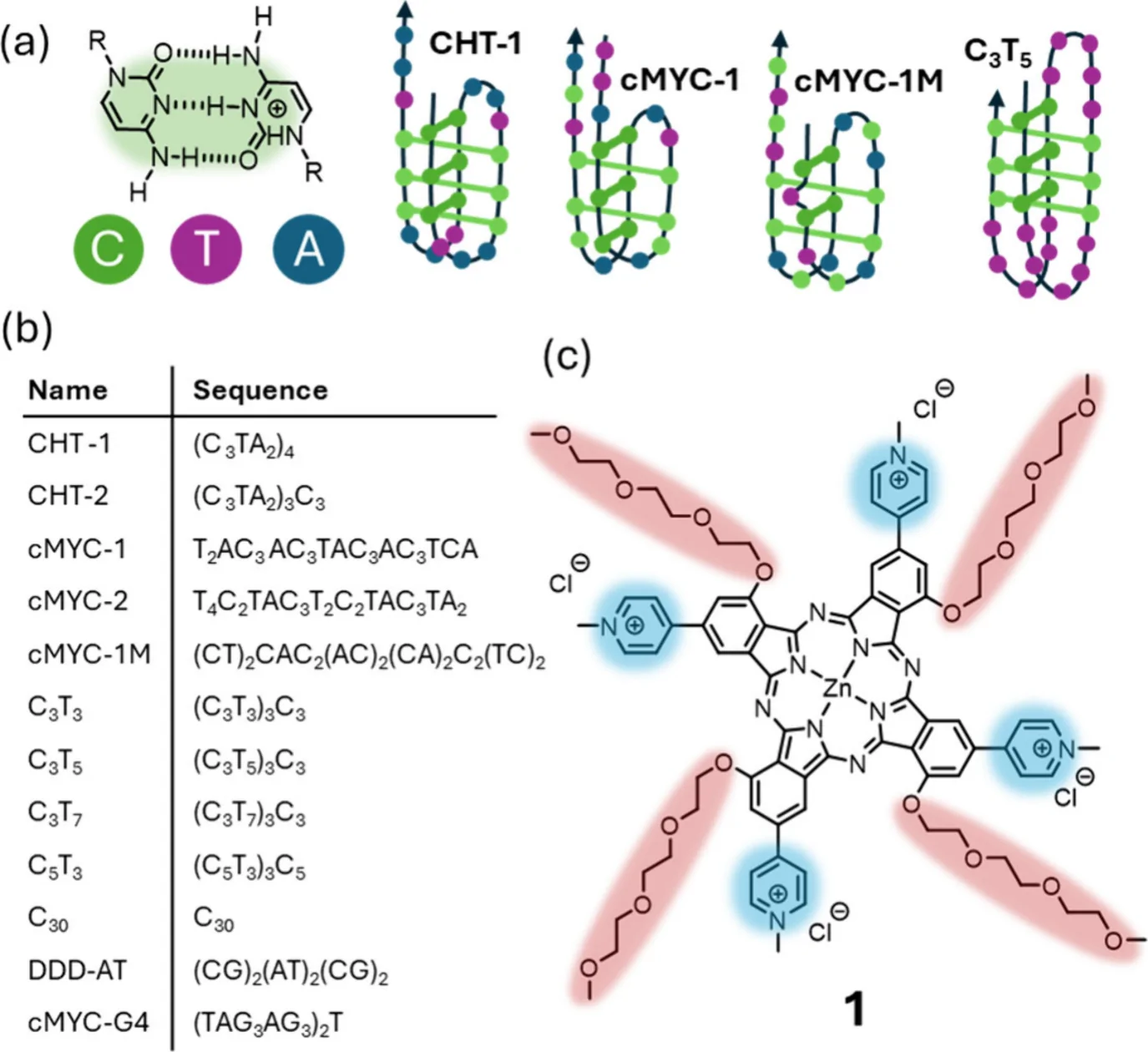
(a) Structure of hemiprotonated cytosine base pair and schematic of i-motif structures. (b) DNA sequences studied. (c) Structure of **1** with substituents highlighted.

Phthalocyanines (Pcs) possess rich photophysics that are highly sensitive to their aggregation state. In the case of ZnPcs, aggregation causes a dramatic pivot from a fluorescent monomer capable of sensitizing singlet oxygen suitable for photodynamic therapy (PDT) to a nonluminescent heat-releasing form with potential application in photothermal therapy (PTT).^14–16^ Pcs bind strongly to DNA, particularly G4 DNA.^17–21^ However, to date no Pcs have been reported to bind iM structures. Recently we reported the ability of guanine-rich sequences to effectively disrupt aggregates of a regiopure *C*_4h_ ZnPc modified with triethylene glycol methyl ether and *N*-methyl-4-pyridinium substituents (**1**) (Figure 1c and Figures S1 and S2).^22^ The dispersion of **1** was linked to the formation of symmetry-matched G4 structures with highly effective disruption observed for the **G-HT** hybrid-G4. Now we report the unexpected ability of iM-forming sequences to disperse aggregates of **1** exclusively in their folded state and to release **1** when the iM is unfolded at increased pH, a distinctly different behavior than observed with G-quadruplex-forming sequences. The iM-forming sequences studied (Figure 1b and Table S1) include variations of biologically relevant cytosine-rich human telomere (**CHT**) and **cMYC** oncogene promoter sequences, model sequences with different cytosine tract and thymine loop lengths (**C**_**x**_ **T**_**y**_) and the **C**_**30**_ sequence that forms an iM at neutral pH (Figures S3 and S4). We also compare binding to a model double-stranded system **DDD-AT** and the G4-forming **cMYC-G4**.

Pc **1** exists primarily as a nonemitting aggregate in aqueous solution between pH 4 – 8.5, characterized by absorbance at 687 nm (Figure S5a).^22^ This pH range is relevant to iM sequences, as they are typically folded at pH 5.5 and unfolded at pH 8.5. A weak 748 nm emission is attributed to a small monomer population, as confirmed by excitation spectra (Figure S5b,c).

The addition of **CHT-1** iM to a solution of aggregated **1** at pH 5.5 resulted in a decrease in the absorbance of **1** at 687 nm and increase at 743 nm (Figure 2a). At just 1.5 equiv of **CHT-1**, a Q-band profile characteristic of purely monomeric **1** was obtained, accompanied by a significant (×20) emission intensity increase (Figure 2b). An absorption redshift from 748 to 755 nm (124 cm^–1^) and a decrease in the band intensity at 330 nm (Figure S6) versus unbound monomer in DMSO were also observed, which reflect environmental changes upon binding to **CHT-1**. In contrast, minimal changes in the absorption and emission spectra were observed upon addition of unfolded **CHT-1** at pH 7.0 and 8.5 (Figure 2c and Figure S7) with **1** remaining aggregated. A slight emission enhancement at pH 7.0 is attributed to a small amount of electrostatic interaction with the single-stranded sequence.

**Figure 2:**
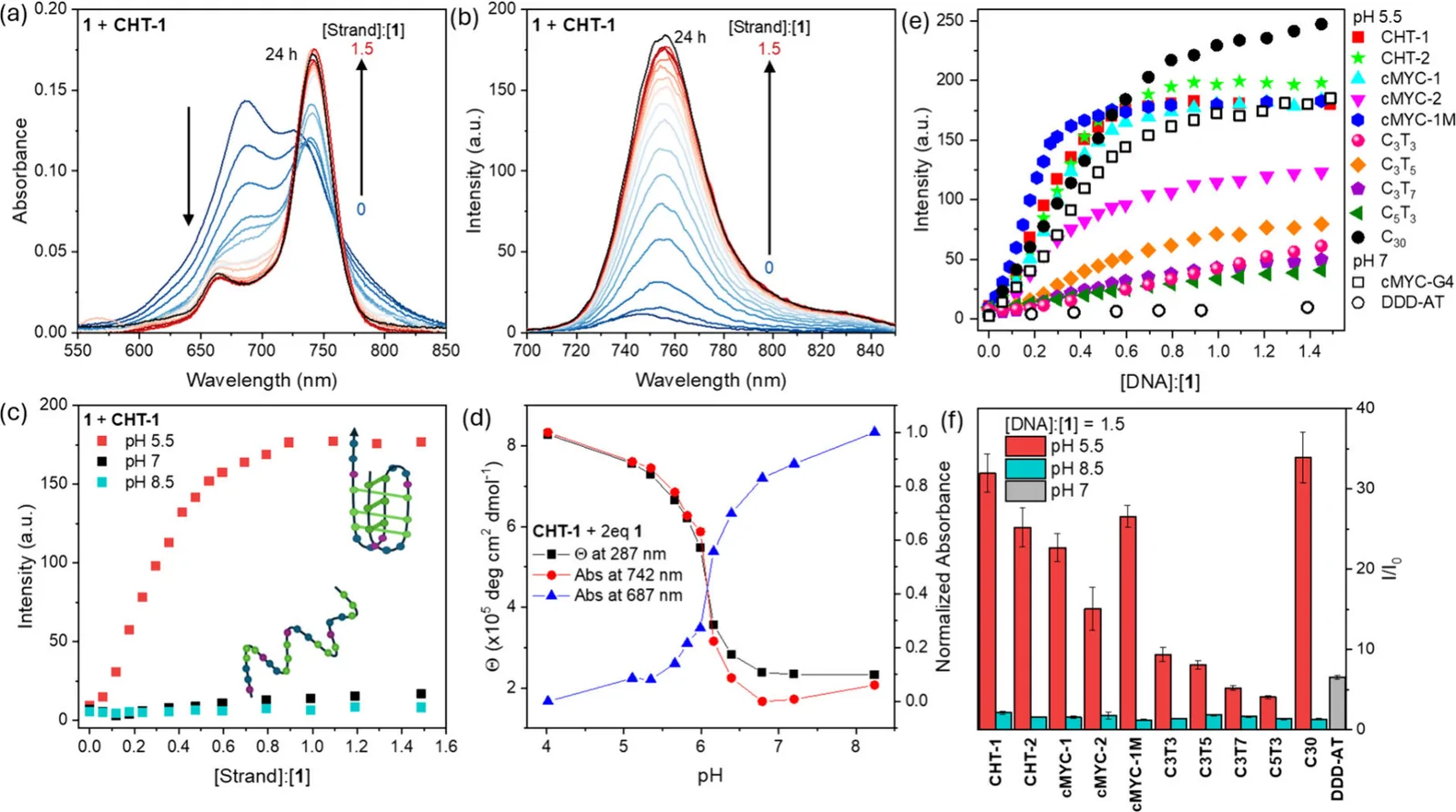
(a) UV–visible absorption and (b) emission spectra of **1** (2.3 μM) with **CHT-1** (0–3.2 μM/strand) at pH 5.5. (c) pH-dependent emission intensity of **1** with **CHT-1**. (d) Molar ellipticity of **CHT-1** (3.3 μM) at 287 nm with **1** (7 μM) and normalized absorbance of **1**. (e) Emission intensity of **1** with iMs at pH 5.5 (colored) and with **cMYC-G4** and double-stranded **DDD**-**AT** at pH 7 with 100 mM KCl (white) against [DNA]:[**1**]. (f) Emission enhancement (*n* = 3) of **1** (2.3 μM) following 24 h incubation with iM sequences (3.2 μM) at pH 5.5 (red) and 8.5 (blue) and **DDD**-**AT** (3.2 μM/duplex, pH 7.0). Recorded in 50 mM potassium phosphate buffer at RT and λ_ex_ = 650 nm.

A Hill plot was used to fit the change in emission intensity of **1** (2.3 μM) with increasing concentration of **CHT-1** at pH 5.5, which gave an apparent half saturation concentration (*K*_app_) of 570 nM with a Hill Coefficient (*n*) of 2.6 (Figure S8a). This indicates a high affinity for **1** and positive cooperativity in the interaction.

Notably, at a fixed [**CHT-1**]:[**1**] ratio of 1:2, a decrease in the pH was accompanied by (i) an increase in the CD molar ellipticity (287 nm) demonstrating **CHT-1** iM formation, (ii) a decrease in the Pc aggregate absorbance (687 nm) and (iii) a concomitant increase in the monomer absorbance (742 nm) (Figures 2d and S9). These observations importantly confirm that aggregate disruption occurs only as the iM folds. **CHT-1** folded cooperatively in the absence of **1**, as observed by a sharp increase in CD signal with decreasing pH (Figure S10).^23^ However, in the presence of **1**, the CD intensity increased over a wider pH range indicating that intermediate folded states may facilitate binding.

The emission intensity as a function of [iM]:[**1**] ratio for all sequences is shown in Figures 2e and S11-S19. Spectral changes similar to those observed for **CHT-1** upon titration with **1** are also seen with **CHT-2** and **cMYC-1** at pH 5.5 with a *K*_app_ of 670 and 700 nM respectively (Figure S8 and Table S2). This suggests that the terminal 5′-TTA-3′ of **CHT-1** is not required for binding to **1**. In contrast, the **cMYC-2** iM-forming sequence^24^ was found to be less effective at disrupting aggregates of **1**. The iMs formed from **C**_**3**_ **T**_**3**_, **C**_**3**_ **T**_**5**_, **C**_**3**_ **T**_**7**_, and **C**_**5**_ **T**_**3**_, which exclusively contain thymine loops, showed limited ability to disrupt aggregates. Changes in loop composition impact stability^25^ with adenine bases in the loops being associated with greater flexibility and lower stability than analogous sequences with thymine. The replacement of TAA loops of **CHT-2** with TTT has been shown to increase stability^23^ and to impact conformation.^26^ This may indicate that **1** requires a more flexible iM scaffold to bind effectively. The greatest intensity response was observed for the **C**_**30**_ sequence, which can form iMs at neutral pH.^27^ Intriguingly, the **cMYC-1M** iM was highly effective at disrupting aggregates of **1** (*K*_app_ = 410 nM). **cMYC-1M** has the same base composition as **cMYC-1** but with a rearranged order leading to shorter cytosine tracts. The CD spectrum is indicative of partial iM formation (Figure S20) and this may facilitate binding. This supports the observation that flexibility may be important for binding of **1**.

A survey of emission enhancement after 24 h incubation with 1.5 equiv of the iM-forming sequences at pH 5.5 and 8.5 is shown in Figure 2f (spectra in Figure S21). In all cases, enhanced emission is observed at iM-forming pH 5.5 compared to pH 8.5. Considerable emission enhancement (×20–35) is observed for **CHT-1**, **CHT-2**, **cMYC-1**, **cMYC-1M** and **C**_**30**_, with differences in the emission intensity attributed to differing binding site environments. In contrast to the time-dependent interactions observed in the presence of guanine-rich nucleic acids,^22^ modest increases in emission intensity are observed with the iMs over time, which can be correlated to the flexibility of the structures (Table S3).

Significantly, a greater emission enhancement of **1** is found in the presence of iMs than in the presence of the double-stranded **DDD-AT**. No emission enhancement was observed in the presence of deoxycytidine monophosphate (dCMP) or the double-stranded {d­(GC)_6_}_2_ at pH 5.5, which further indicates the role of the iM structure (Figure S22). Impressively, the ability of the **CHT-1**, **CHT-2** and **cMYC-1** iMs to disrupt aggregates of **1** is comparable to that of the parallel quadruplex, **cMYC-G4** (Figure 2e). This is all the more striking as **cMYC-G4** is more effective at disrupting aggregates of **1** than the **G-HT** hybrid quadruplex (Figure S23).^22^

The addition of **1** to solutions of **CHT-2** and **cMYC-1** at pH 5.5 caused a decrease of the CD band intensity (287 nm) (Figures S24 and S25). This was particularly significant for **cMYC-1** where continuous decreases were observed up to 8 equiv of **1**. However, the CD band does not red-shift to the equivalent maximum position observed for the unfolded strand at pH 8.5 (Figure S26). Notably, at greater than two equivalents the population of aggregates of **1** significantly increases (Figure S25e). As commented on above, **1** may have a role in loosening or partial unfolding of the iM structure. This has been previously observed for porphyrin structures with iMs.^28^ Additionally, high concentrations of **1** may lead to a condensation event, which also results in a decrease in CD signal.^9,29^ Note, no CD bands were observed for the aggregates or monomers of **1** in the absence of DNA (Figure S27).

For **cMYC-1M**, a slight redshift was observed with no loss in intensity (Figure S24e,f). These observations suggest that the iM structure is largely preserved during binding. The pH-dependent folding of **cMYC-1M** (measured by CD at 282 nm) was found to occur at similar pH in the absence and presence of 2 equiv of **1**, and the transition from aggregate to monomer of **1** occurred at a similar pH (Figure S28), as was observed for **CHT-1**. This again confirms the critical role of the iM structure in aggregate disruption.

Thermal denaturation studies (16 to 95 °C) of the **CHT-1**, **cMYC-1** and **cMYC-1M** iMs in the absence and presence of **1** (0.5–2 equiv) were performed by monitoring the change in the UV absorbance of the iM at 260 and 295 nm, the Pc monomer Q-band (742 nm) and the aggregate band (687 nm). A slight broadening in the UV (260 nm) melting curve of **CHT-1** was observed in the presence of **1** (0.5–2 equiv) without significant change in the denaturation temperature (*T*_M_) of 45 °C (Figure 3a). iM melting was confirmed by CD (Figure 3b) and broadening was observed in the CD melting curve in the presence of **1** (2 equiv) with the loss of an isodichroic point at 275 nm (Figure S29). Thermal denaturation of **CHT-1** is accompanied by a decrease in the monomer Q-band (742 nm) and an increase of the aggregate band (687 nm) (Figure 3c and Figure S30a). This was found to be completely reversible with recovery of the monomer absorption bands following DNA annealing (Figure 3d). Note, no hysteresis was observed in melting/annealing of **CHT-1** (Figure S30b). These results indicate that binding of **1** does not significantly stabilize the **CHT-1** iM, and as it unfolds, **1** is released into solution where it aggregates. Crucially, this aggregation is found to be reversible upon refolding of the iM structure. Thermal denaturation of **cMYC-1** and **cMYC-1M** also resulted in aggregation of **1** but no broadening of the melting curves was observed (Figures S31 and S32).

**Figure 3:**
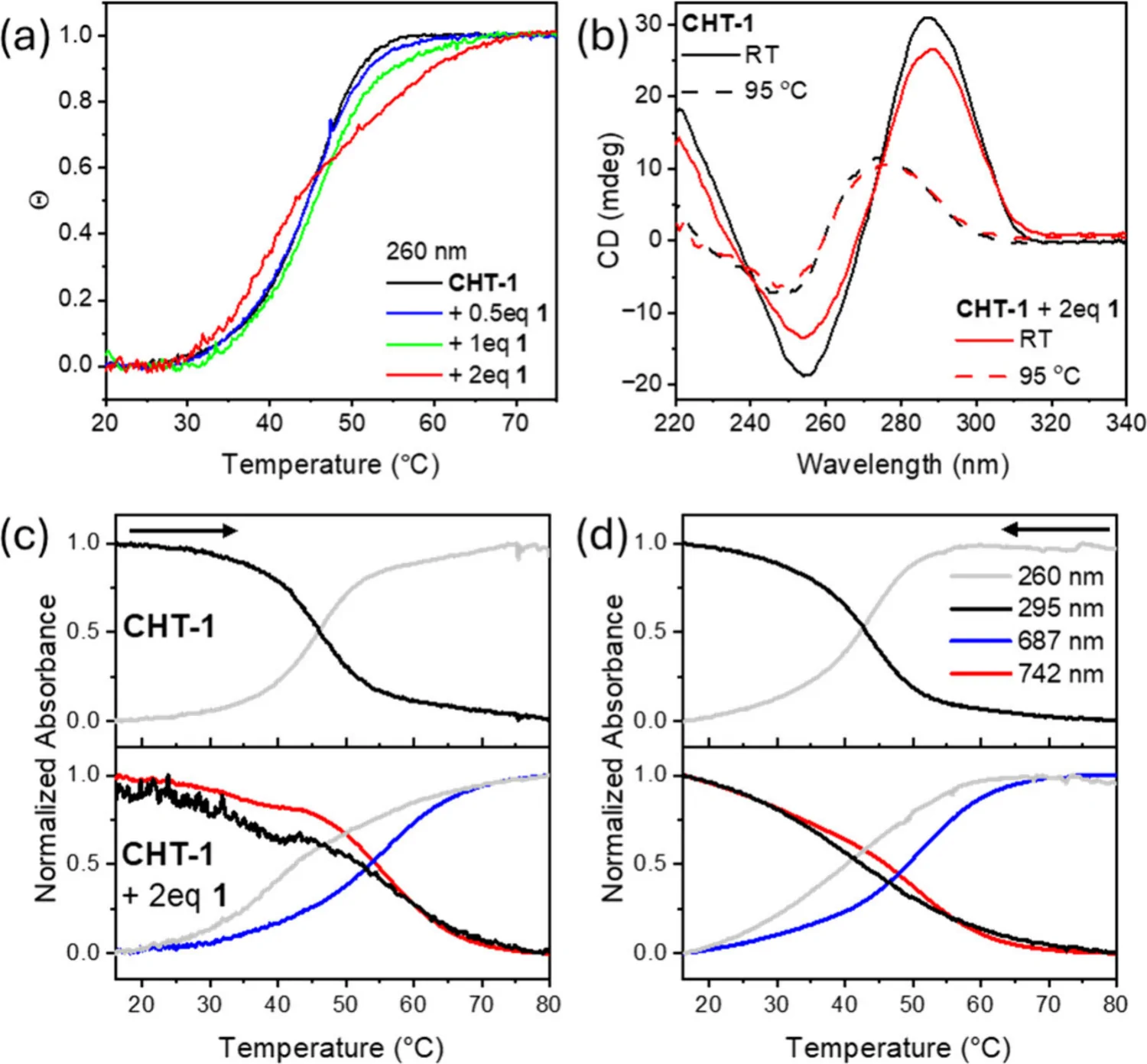
(a) Fraction of unfolded **CHT-1** (1.8 μM) with **1** (0 – 3.6 μM) calculated from UV–visible absorbance at 260 nm against temperature. (b) CD spectra of **CHT-1** (4.2 μM) with **1** (0 – 8.4 μM) at RT and 95 °C. Normalized absorbance of **CHT-1** (1.8 μM) at 260 and 295 nm and **1** (3.6 μM) at 687 and 742 nm as a function of temperature when (c) heated and (d) cooled. Recorded in 20 mM sodium acetate buffer. Heating/cooling rates = 1 °C/min.

In the absence of DNA, the aggregates of **1** are slightly disrupted with increasing temperature as observed from an increase in absorbance of the monomer Q-band at 742 nm and decrease of the aggregate band at 687 nm (Figure S33a–c). No significant absorbance change is observed at 260 and 295 nm where iM unfolding is observed. Interestingly, **1** appears to be slightly more aggregated at 95 °C in the presence of DNA compared to in buffered solution alone (Figure S33d), which may be due to additional crowding facilitated by electrostatic interaction with DNA. However, in the absence and presence of DNA at 95 °C, **1** remains primarily aggregated relative to the monomer species observed in the presence of iM DNA at RT.

For **CHT-1** in the presence of **1** (2 equiv), a notable offset of 6.5 °C was observed between *T*_M_ and the aggregation temperature *T*_A_ (where **1** was 50% aggregated). This is observed in both heating and cooling cycles (Figure 3c,d) and increases to 14 °C for 0.5 equiv of **1** (Figure S34 and Table S4). The broadening of the UV and CD melting curves, in addition to the decrease in CD intensity with the complex may indicate a relaxation of the iM structure and a favoring of partially disrupted **CHT-1** for binding **1**.^23^ This may explain the lack of binding to iM structures with thymine-rich loops, the higher stability of these structures may prevent loosening of the structure to accommodate **1**.

Prior NMR structural studies indicate that positively charged TMPyP4 porphyrin^12^ and positively charged cyclic tetraoxazole^30^ molecules participate in end-on binding, while side-on binding has also been predicted by NMR and using molecular modeling.^31–33^ Informed by the spectroscopic studies molecular docking was performed between the optimized structure of **1** (Figure S36) and an intramolecular iM crystal structure (PDB: 8AYG).^34^ Two models of were constructed with 2:1 (**1**:DNA) stoichiometry with either side-on or end-on binding of **1** to the iM structure (Figures S37–S40). The docking experiments reveal a stabilizing role of electrostatic interactions between the positively charged **1** (4+) and negatively charged phosphate groups of the DNA. These interactions are more numerous in the side-on model (*ca* −40 kcal/mol) compared to the end-on model, leading to an energetically more stable structure (Figure S39). Repulsive interactions (*ca* +35 kcal/mol) between the adenine and **1** in the end-on configuration point to the potential role of loop interactions (Figure S38). It is interesting to note that the emission of **1** bound to 1.5 equiv of **CHT-1** is unaffected by the addition of 100 mM KCl, which supports the role of other dispersion interactions (Figure S41).

Having investigated the binding interactions, the impact of the iM structure on the photophysical properties of **1** was further explored. The fluorescence lifetime of 1 in the presence of the iMs ranged from 1.97 ns (**cMYC-1**) to 2.09 ns (**CHT-1**). The average value of 2.0 ns is comparable to that for the monomer in DMSO, which further supports the interaction of discrete monomers with the iM structure (Table S5). The photoluminescence quantum yield (PLQY) of **1** was measured in buffered solution, with one iM structure that showed good aggregate disruption (**cMYC-1**) and in DMSO. The PLQY of **1** in buffered solution was found to be close to 0% while with **cMYC-1** this increased to 5.2% and in DMSO it was found to be 11.9% (Table S6).

Finally, the comparative ability of DNA structures to modulate the biologically relevant activity of **1** was investigated by measuring the generation of photosensitized ^1^O_2_ upon irradiation of the monomer (Figure 4a). Notably, while ^1^O_2_ phosphorescence at 1270 nm was not observed upon excitation of aggregated **1** alone in water or in the presence of the unfolded **CHT-1** or double-stranded **DDD-AT**, it was observed in the presence of the **CHT-1** and **C**_**30**_ folded iMs, indicating turn-on PDT activity may be possible upon iM binding. Reversible ^1^O_2_ production was previously reported for an iM-forming sequence covalently modified with a ^1^O_2_ sensitizer and a quencher at opposites ends that are in close proximity in the iM structure and are separated when the iM unfolds in basic conditions.^35^ In contrast, in this study, the switching on of ^1^O_2_ production is directly triggered by disaggregation of **1** in the presence of a folded iM structure, which does not require modification of the iM-forming sequence.

**Figure 4:**
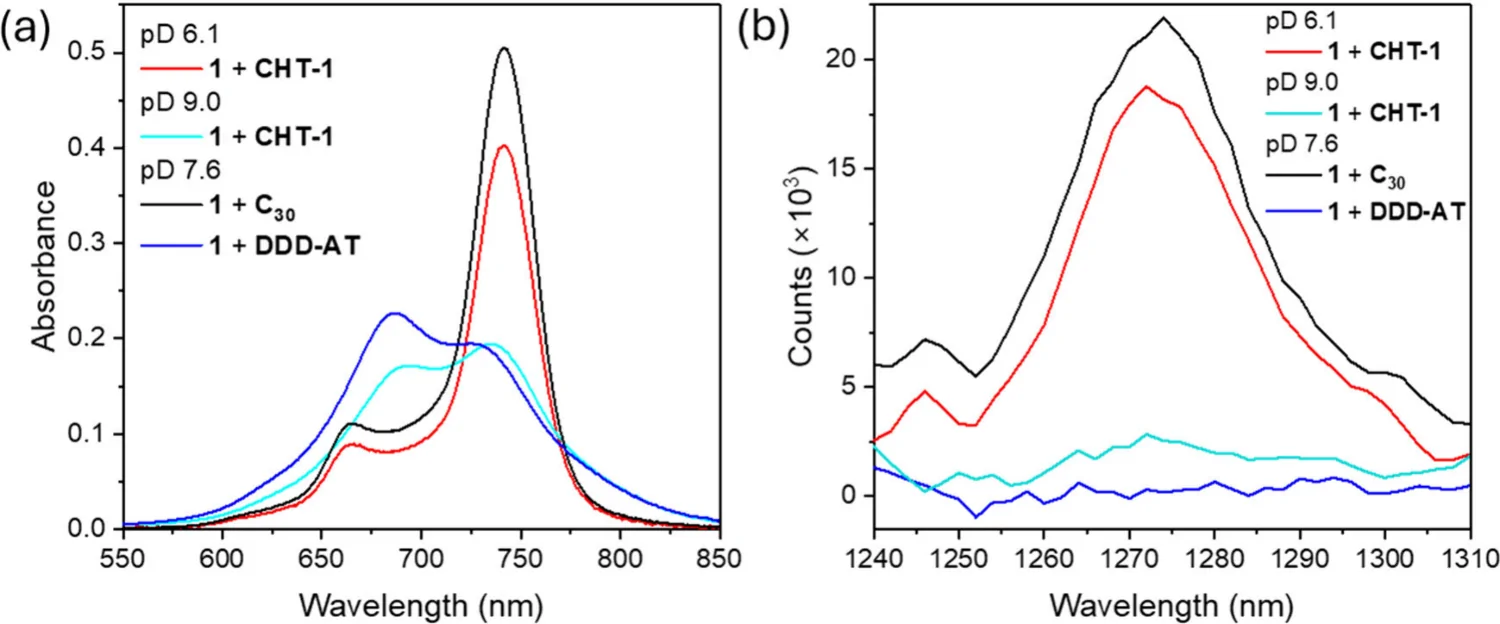
(a) UV–visible absorption spectra of **1** (4.6 μM) with **CHT-1**, **C**_**30**_ (4.6 μM/strand), and **DDD-AT** (4.6 μM/duplex) in buffered D_2_O (50 mM potassium phosphate buffer) at pD 6.1, 7.6, 9.0. (b) Emission spectra of ^1^O_2_ following excitation of **1** (λ_ex_ = 400 nm). The iM structure of **CHT-1** under these conditions was confirmed using CD spectroscopy (Figure S35).

To conclude, the results of this study reveal the ability of the folded iM structure to effectively disperse aggregates of **1**, which results in switching on its NIR emission and ^1^O_2_ photosensitization. The observation that the dispersion of photosensitizer **1** can be reversibly triggered by temperature and pH (Figure 5) has implications for the potential cellular activity of **1**. The aggregate PTT activity is expected to be replaced by PDT processes in the presence of iM-forming DNA at suitable pH and temperature. This phenomenon is somewhat related to the growing interest in DNA drug delivery by systems including DNA origami^36,37^ and the recent report of pH-triggered doxorubicin release from iM-modified liposomes.^38^ The solution studies suggest that flexibility in the iM structure facilitates dispersion of **1**, and the denaturation experiments indicate some association of **1** to a partially folded structure, see proposed mechanism in Figure 5c. The unusual preference for iM over double-stranded DNA is attributed to the unique molecular structure of **1** whose pyridinium units allow for electrostatic interactions while the Pc core provides a rigid binding platform. Additionally, the inclusion of the triethylene glycol unit may introduce steric factors that favor binding and provide a favorable local binding environment at the iM structure, as related PEG molecules are known to stabilize iM structures.^39^ This will be the subject of future research.

**Figure 5:**
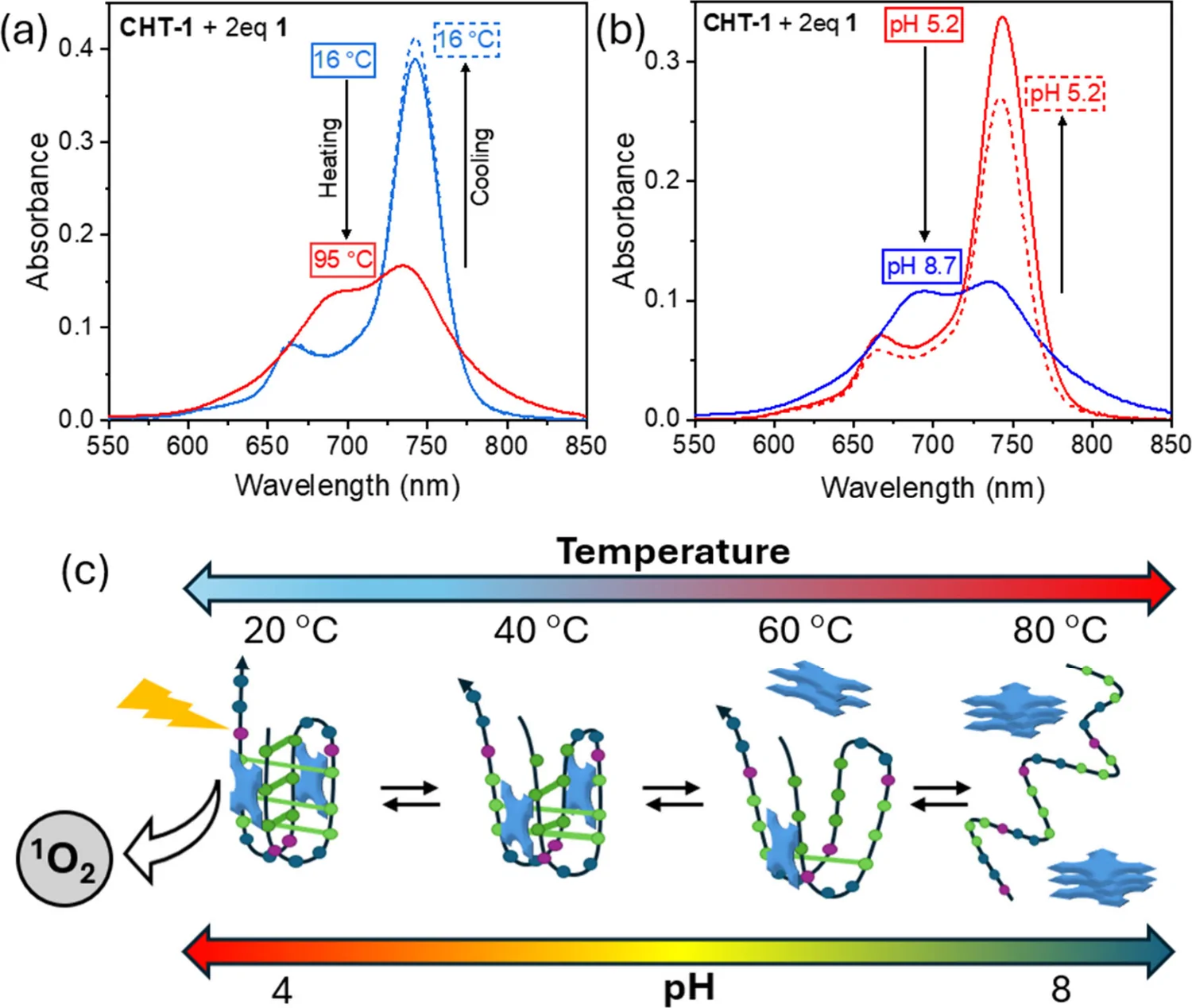
UV–visible absorption spectra of **1** (3.6 μM) with **CHT-1** (1.8 μM) with varying (a) temperature in 20 mM sodium acetate buffer, pH 5.5 (heating/cooling rate = 1 °C/min) and (b) pH in 50 mM potassium phosphate buffer at RT. Solid lines at 16 °C and pH 5.2 indicate absorption prior to iM unfolding and dashed lines indicate absorption following iM refolding. (c) Schematic of possible binding interaction informed by docking experiments, iM unfolding and release of **1**.

## Supplementary Material



## Notes

Materials and methods, supplementary spectroscopic and molecular modeling data, and supporting tables of spectroscopic changes (PDF)
