# Supplementary material for: DNA i‑Motifs Reversibly Disperse and Modulate the Photophysical Properties of an Aggregated Zinc Phthalocyanine Photosensitizer, Triggered by pH and Temperature
Source: J Phys Chem Lett. 2026 Sep 7;17(37):10570–6. doi: 10.1021/acs.jpclett.6c01427 (PMC13592073; doi:10.1021/acs.jpclett.6c01427)
Supplement: Supplementary file 1 [file jz6c01427_si_001.pdf]

Supporting Information

DNA i-Motifs Reversibly Disperse and Modulate the Photophysical Properties of an Aggregated Zinc Phthalocyanine Photosensitizer, Triggered by pH and Temperature

Eleanor R. Windle,[a] Nadia Elghobashi-Meinhardt,[a] Robert M. Edkins,*[b] and Susan J. Quinn *[a]

a. School of Chemistry, University College Dublin, Dublin 4, Ireland, D04 V1W8.

b. Department of Pure and Applied Chemistry, University of Strathclyde, Glasgow, UK, G1 1XL.

Email: susan.quinn@ucd.ie and robert.edkins@strath.ac.uk

Table of contents **Page**

S1 Experimental S2

S2 Figures S4

S3 Tables S39

S4 References S41

S1 Experimental

Materials:

The DNA sequences used (Table S1) were synthesized and desalted by Eurogentec and were used as received. Deionized water was obtained from an ultra-pure Millipore filtration system (1.82 mΩ). **1** was synthesized as previously reported by us.[1] An anion exchange of **1** from the iodide to the chloride salt was performed by gently shaking a methanol solution of **1** with Amberlite exchange resin (IRA402, Cl form, prewashed with methanol) for 2 h. Following filtration of the resin, the methanol solvent was removed by rotary evaporation at 40 °C. This procedure was repeated three times. ^{1}H NMR spectroscopy was used to assess that the anion-exchange process had not degraded the phthalocyanine (see Figure S1). The anion exchange from iodide to chloride does not materially change the photophysical properties of **1**, as we will report elsewhere.[2]

Instrumentation and Methods

UV-visible absorption spectra were recorded at room temperature (RT) using an Agilent Cary 50 spectrophotometer (Cary WinUV software) using background correction in a quartz cuvette (path length = 1 cm, unless otherwise stated) with a scan speed of 600 nm/min. Emission spectra were recorded on a Varian Cary Eclipse (Cary Scan software) with an excitation wavelength of 650 nm and 10 nm slit widths for both excitation and emission. Time-correlated single-photon counting fluorescence lifetime measurements were recorded on an Edinburgh Instruments FS5 Spectrofluorometer with a 450 nm picosecond pulsed diode laser and the recorded data were fitted using reconvolution analysis (Fluoracle software) of a single exponential decay function and the instrument response function measured via scattering of a Ludox solution. Phosphorescence measurements were performed on an Edinburgh Instruments FLS1000 spectrometer equipped with a liquid nitrogen– cooled, NIR-sensitive InGaAs photomultiplier tube. All spectra were fully corrected for the spectral response of the respective instrument. Circular dichroism (CD) spectra were recorded on a Jasco J-810 spectropolarimeter between 400 – 200 nm with a scanning speed of 100 nm/min, averaged over 8 scans. pH was recorded using a Mettler-Toledo micro pH electrode.

Nucleic acid concentrations were determined spectrophotometrically using the extinction coefficients in Tables S1. Concentrated **1** stock was prepared by dissolving in DMSO. The concentration was determined spectrophotometrically (ε_{737} = 1.74 x 10^5 M^{-1} cm^{-1}) in DMSO. DNA titrations were performed by recording the UV-visible absorption spectrum and emission spectrum of **1** (2.3 µM) following successive addition of aliquots of DNA (3 - 5 µL aliquots of 42 µM stock, final [DNA] = 3.2 µM) in 50 mM potassium phosphate buffer (pH 5.5, 7, or 8.5).

The results are shown as a ratio of DNA structure (e.g. strand, duplex, iM, or G4) to complex concentration ([DNA]:[**1**]). DNA incubation experiments were performed by addition of a single DNA aliquot to **1** (2.3 μM) followed by 24 h incubation in the dark following which UV-visible absorption and emission spectra were recorded. CD titrations were performed with an initial DNA solution (with maximum absorbance of 0.8) in 50 mM potassium phosphate buffer followed by successive addition of aliquots of **1** (5-10 μL). Samples were allowed to equilibrate for 2 h prior to recording of the CD and UV-visible absorption spectra. Variable pH studies were conducted by gradual addition of 0.5 – 2 M HCl or 2 M NaOH to a solution of **1** and DNA in a cuvette and direct pH measurement using a micro pH electrode followed by recording the CD and UV-visible spectra.

Absorption thermal denaturation experiments were performed using a Cary 3500 UV-vis spectrophotometer. The cell temperature was ramped from 16 °C to 95 °C (1 °C/min) as recorded by internal temperature probes and the absorbance was recorded at 260, 295, 687, 742, 900 nm every 0.2 °C. CD thermal denaturation experiments were performed using a JASCO J-810 spectropolarimeter with an external Peltier unit that was ramped between 20 °C and 95 °C at a rate of 1 °C/min. The intensity at 288 nm was recorded every 0.2 °C and the spectrum was recorded between 220 nm and 320 nm at 5 °C intervals. All thermal denaturation experiments were conducted in 20 mM sodium acetate buffer.

Photoluminescence quantum yields were measured using an Edinburgh Instruments FS5 Spectrofluorometer equipped with an SC-30 integrating sphere module. Solution samples and the corresponding solvent blanks were measured in a 1 cm quartz cuvette using direct excitation (λ_{ex} = 680 nm) and the absolute quantum yield was calculated in Fluoracle using the Quantum Yield Wizard.

Hill plot. Titration data of change in emission of **1** ($I - I_0$) against [DNA] were fit using a Hill Fit (Equation 1) on Origin Pro labs.

$$I - I_0 = \frac{(I - I_0) * [DNA]^n}{(K_{app})^n + [DNA]^n} \quad (1)$$

pD values were estimated by recording solutions using a pH meter to obtain pH* and using Equation 2 to calculate pD.[3]

$$pD = pH^* + 0.4 \quad (2)$$

Molecular Modelling

A review of the Protein Data Bank (RCSB.org)[4] reveals relatively few solved structures for the cytosine i-motif and none matching the exact systems in this paper. Therefore, docking experiments were performed on the intramolecular i-motif formed from the TATCCCCACACCCCTATCCCCACACCCCTAT sequence that is located at the insulin-linked polymorphic region (ILPR) (PDB:8AYG) (2.25 Å).[5]

The xyz-structural data of zinc phthalocyanine **1** without counter anions was generated using Avogadro 1.2.0.[6] The charge distribution of **1**, assuming an overall charge of +4, was calculated with Gaussian16[7] at B3LYP/6-31g(d) level of theory, with Grimme empirical dispersion.[8] Force field parameters of **1** with charge +4 were generated using the Cgenff server[9] without Zn^{2+}. Next, Zn^{2+} was manually reinserted and the charges on coordinating nitrogen atoms were adjusted according to the Mulliken charge assignment to maintain an overall charge of +4 (Figure S35).

Informed by the spectroscopic titrations two models of **1**:DNA (2:1 stoichiometry) binding interactions were constructed that located **1** with either side-on or end-on binding to the iM structure (Figure S36). This was done manually using VMD (version 1.9.4a57)[10]. The final molecular structure was then built using the CHARMM-GUI server,[11] with the CHARMM36 all-atom parameters for nucleic acid and ions,[12] as implemented in CHARMM 47a2,[13] and considering an aqueous environment with a 0.05 M K_3PO_4 buffer solution. Each **1**:DNA system was solvated in a rectangular box with explicit TIP3 water[14] and K^+ and PO_4^{3-} ions, corresponding to 0.05 M K_3PO_4 (Figure S37). The final solvated model consists of ~25000 atoms in a cubic box (length of side 64 Å).

S2 Figures

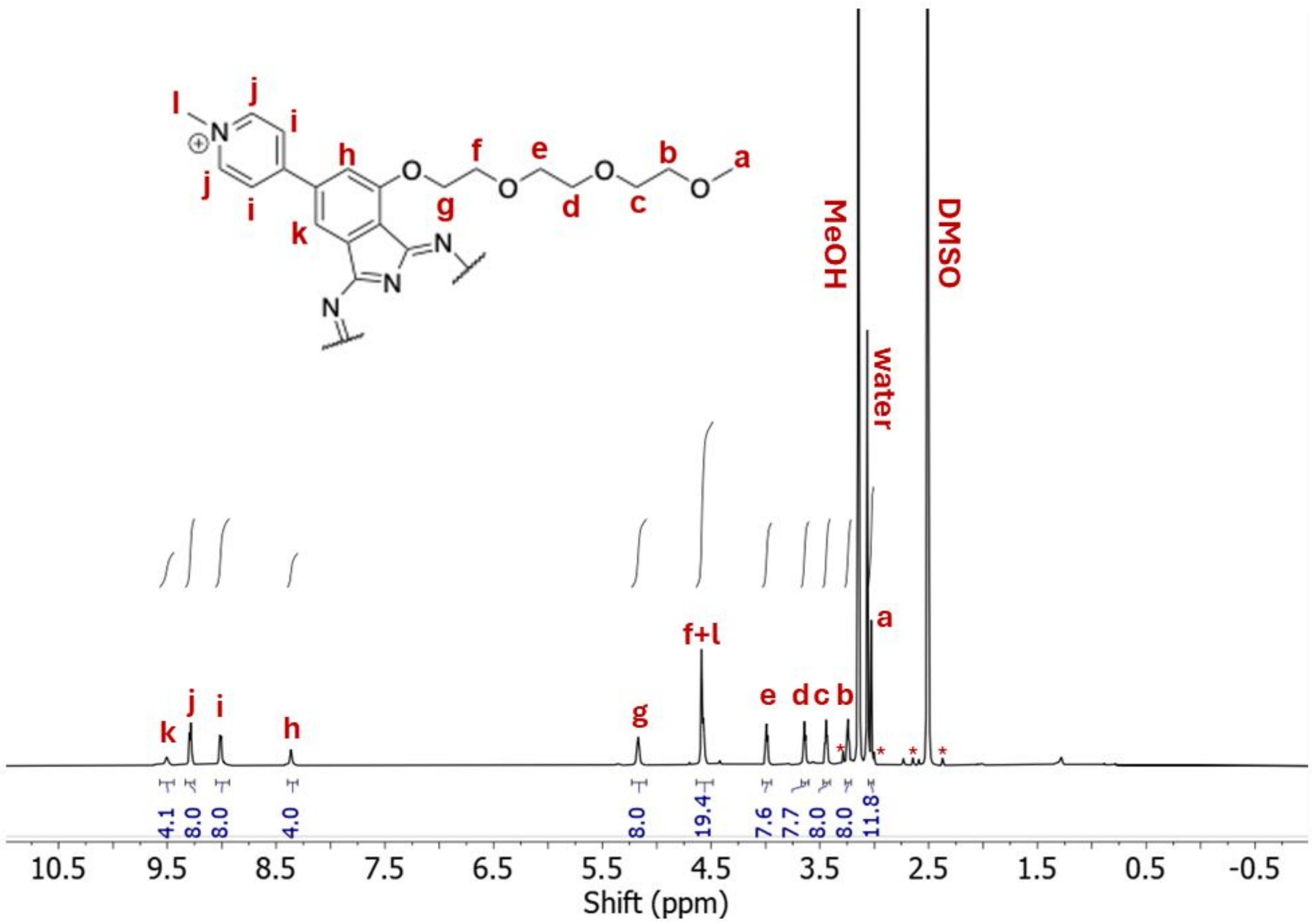

Figure S1. ^{1}H NMR spectrum (DMSO-d6, 500 MHz) of **1** following anion exchange from iodide to chloride salt. * = ^{13}C satellite of solvent.

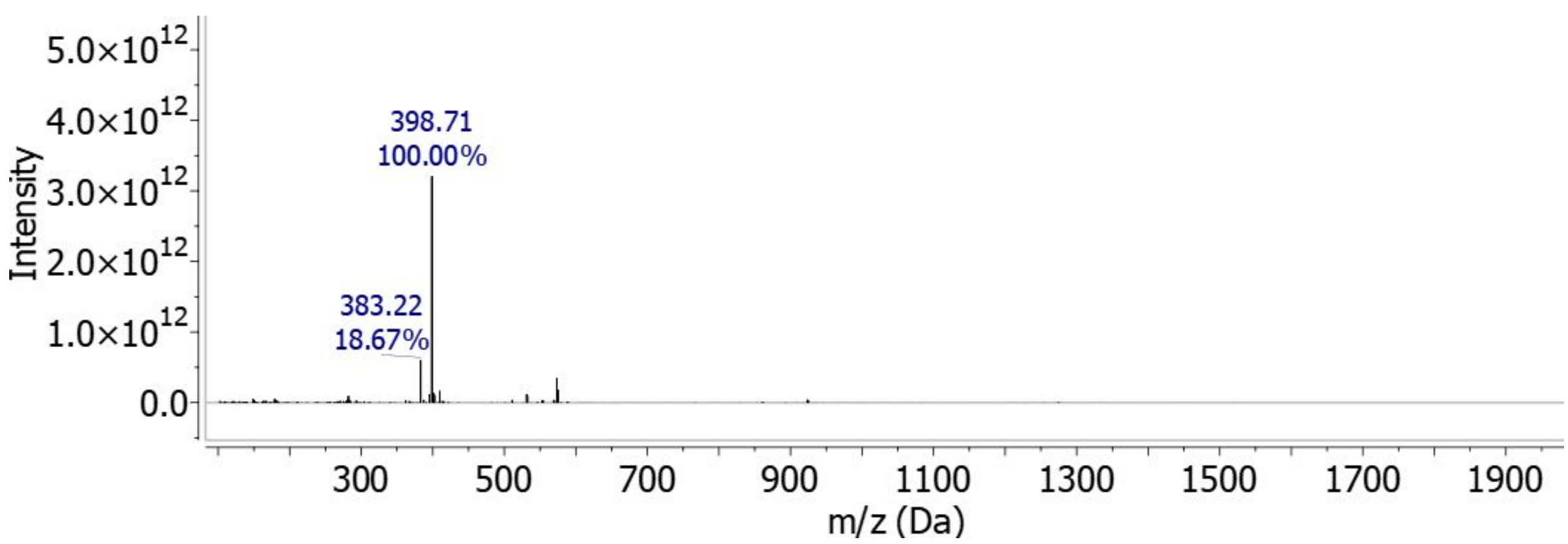

Figure S2. ESI^{+} mass spectrum of **1** (direct injection, total ion spectrum).

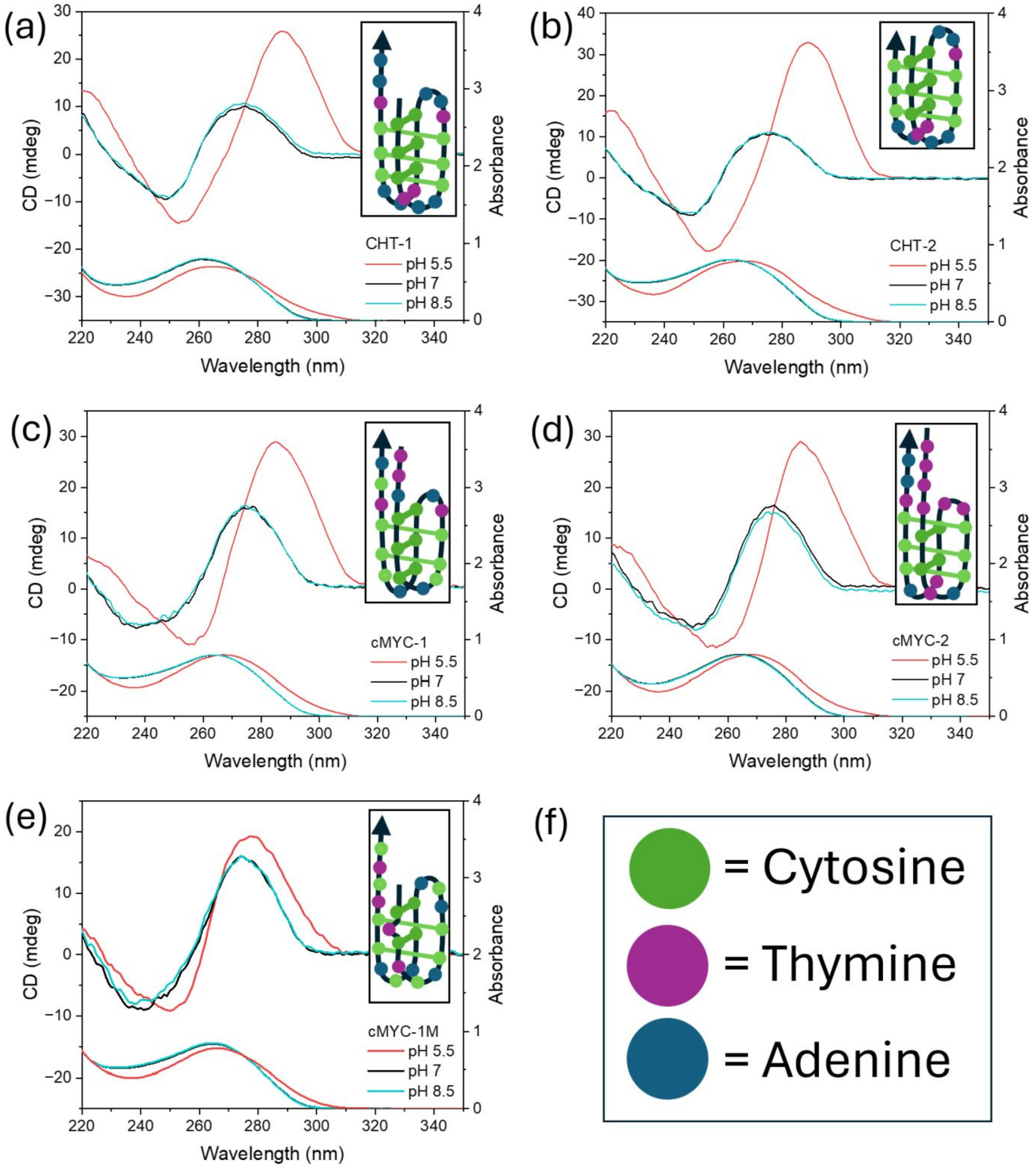

Figure S3. CD (upper data, left axis) and UV-visible absorption (lower data, right axis) spectra of (a) **CHT-1** (3.6 μM), (b) **CHT-2** (4.3 μM at pH ≥ 7, 5.0 μM at pH 5.5), (c) **cMYC-1** (4.2 μM at pH ≥ 7, 4.8 μM at pH 5.5), (d) **cMYC-2** (4.0 μM at pH ≥ 7, 4.6 μM at pH 5.5), and (e) **cMYC-1M** (4.2 μM) at pH 5.5, 7.0 and 8.5 in 50 mM potassium phosphate buffer at RT. Insets show schematics of respective iM structures. (f) Base colour assignment of structural schematics.

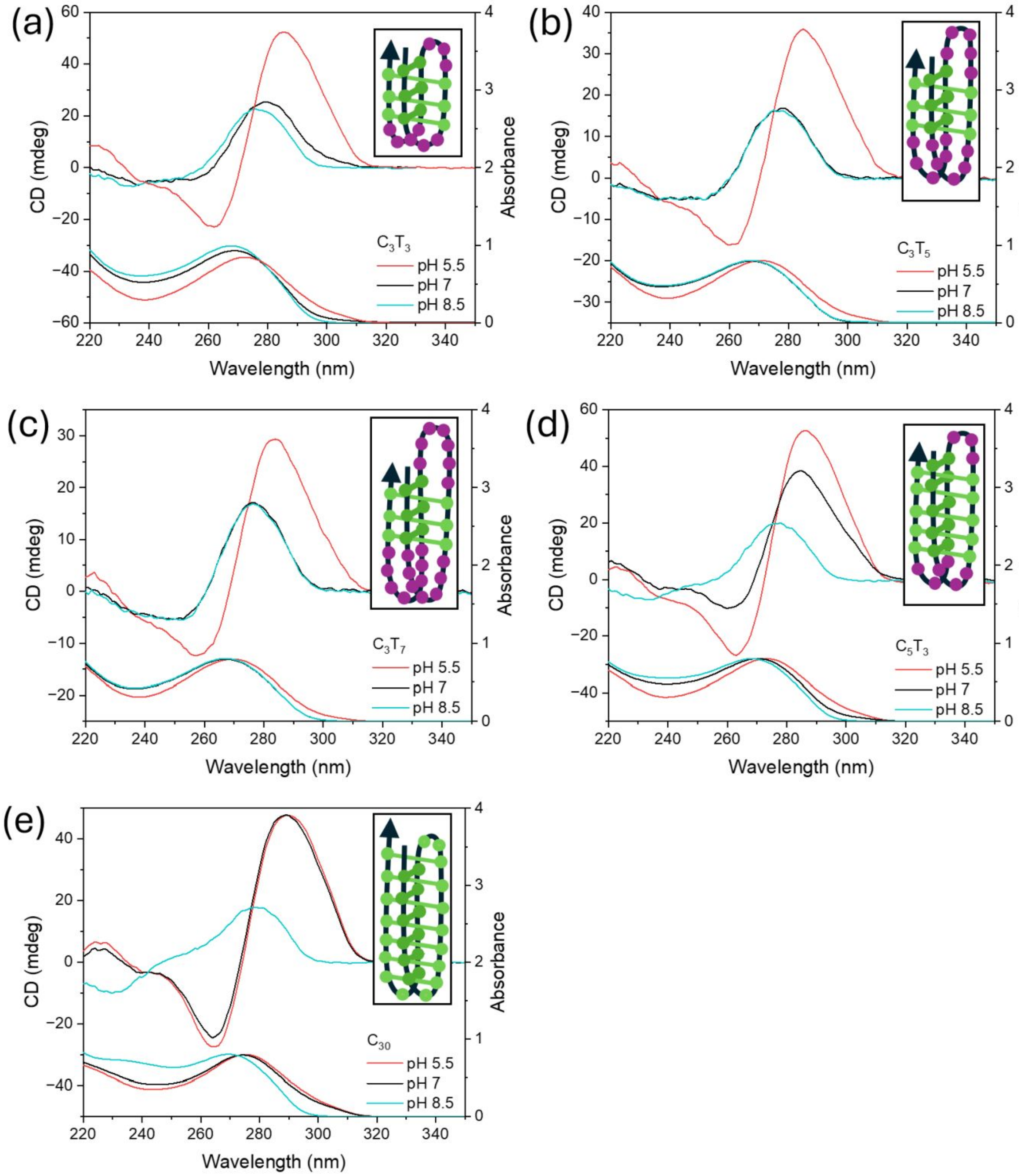

Figure S4. CD (upper data, left axis) and UV-visible absorption (lower data, right axis) spectra of (a) **C_3T_3** (6.2 µM), (b) **C_3T_5** (3.9 µM at pH ≥ 7, 4.4 µM at pH 5.5), (c) **C_3T_7** (3.1 µM at pH ≥ 7, 3.6 µM at pH 5.5), (d) **C_5T_3** (4.0 µM at pH 8.5, 4.4 µM at pH 7, 4.6 µM at pH 5.5, (e) **C_{30}** (3.7 µM at pH 8.5 and 4.0 µM at pH 5.5 and 7) at pH 5.5, 7.0, and 8.5 in 50 mM potassium phosphate buffer at RT. Insets show schematics of respective iM structures.

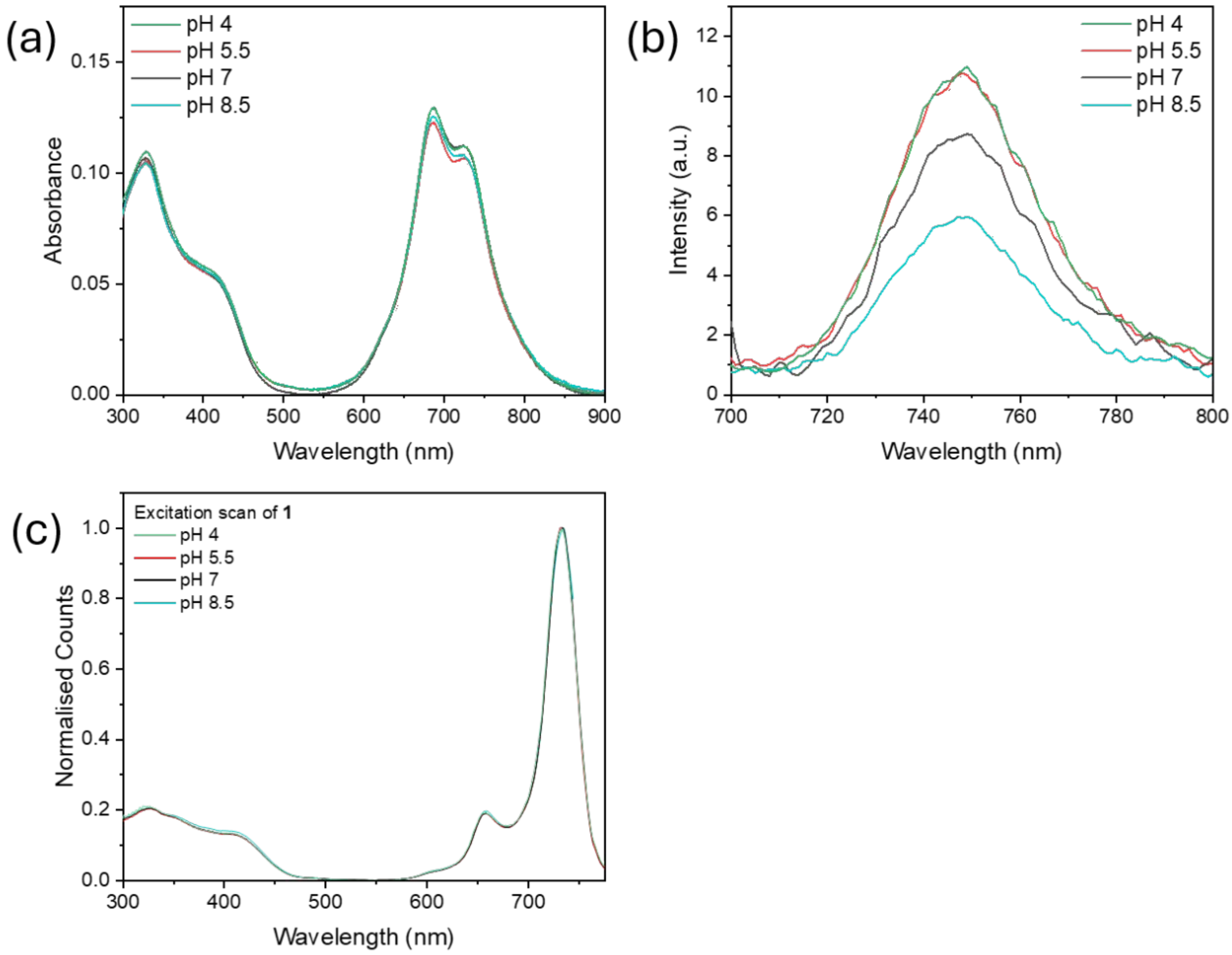

Figure S5. (a) UV-visible absorption, (b) photoluminescence emission, and (c) normalised excitation spectra (λ_{ex} = 650 nm, λ_{em} = 825 nm) of **1** (2.3 μM) at pH 4, 5.5, 7, and 8.5. All spectra were recorded in 50 mM potassium phosphate buffer at RT.

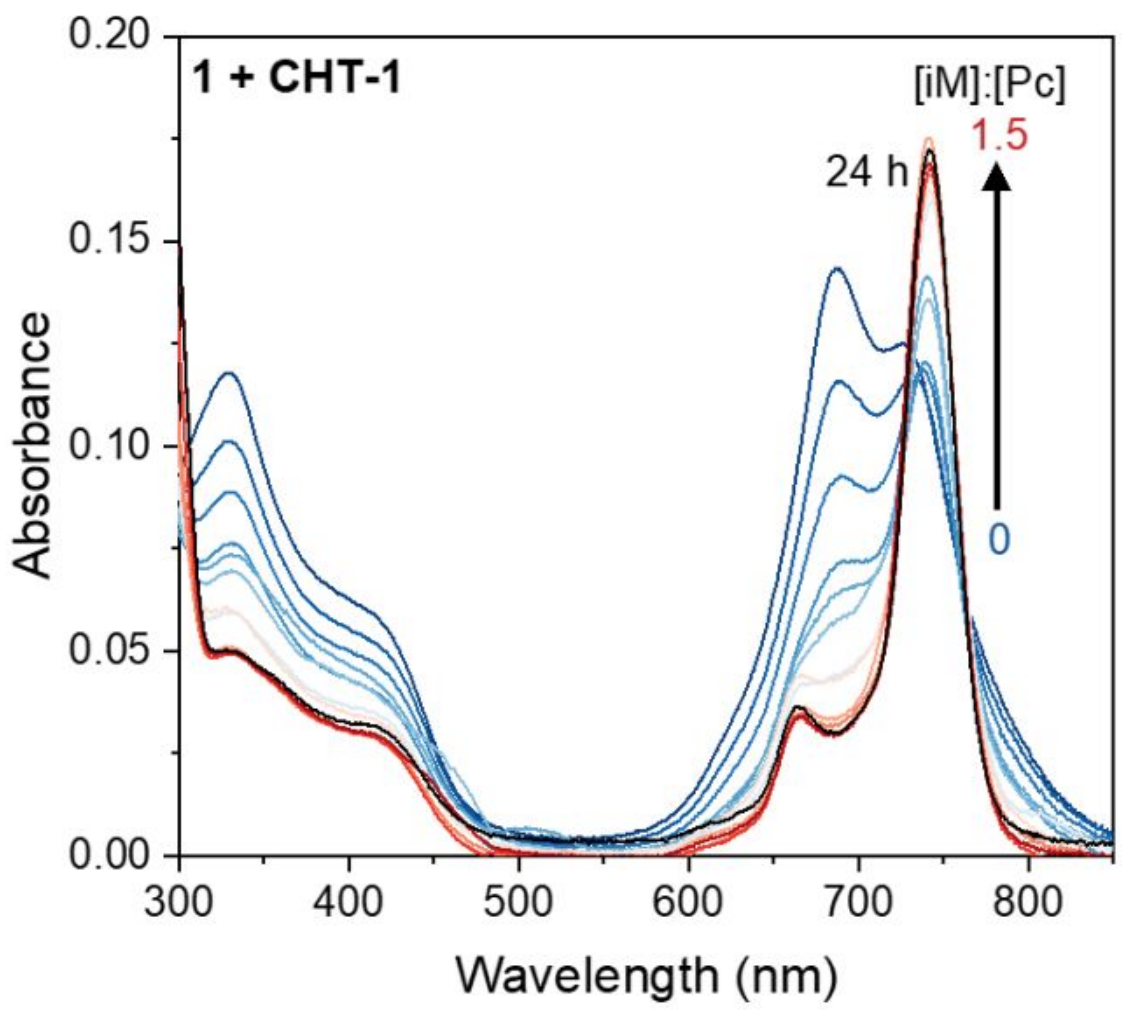

Figure S6. UV-visible absorption spectra of **1** (2.3 μM) titrated against increasing concentration of **CHT-1** (0-3.2 μM strand) in 50 mM potassium phosphate buffer (pH 5.5) at RT.

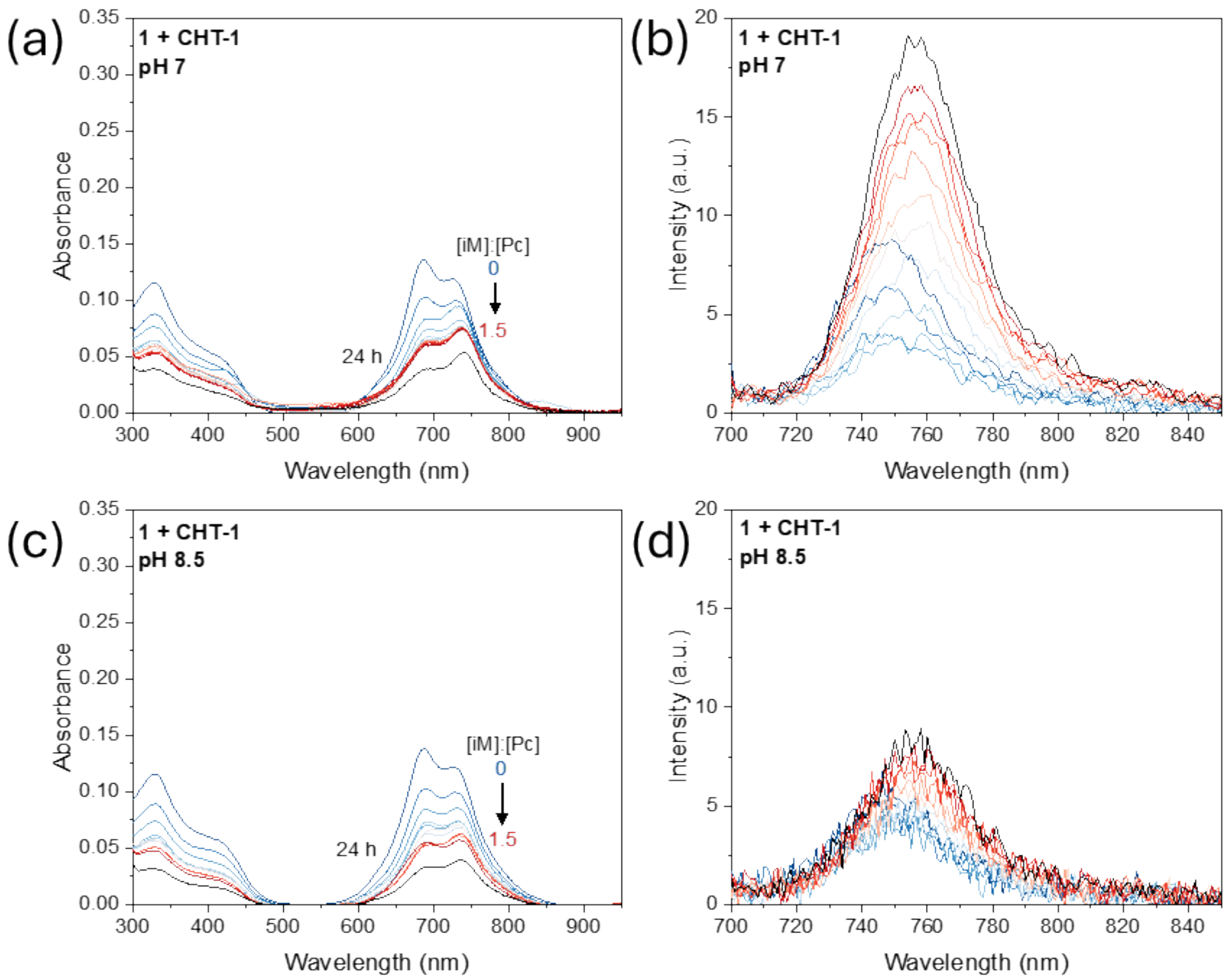

Figure S7. UV-visible absorption (left) and emission (right, λ_{ex} = 650 nm) spectra of **1** (2.3 μM) titrated against increasing concentration of **CHT-1** (0-3.2 μM strand) at pH 7 (a, b) and pH 8.5 (c, d). All spectra were recorded in 50 mM potassium phosphate buffer at RT.

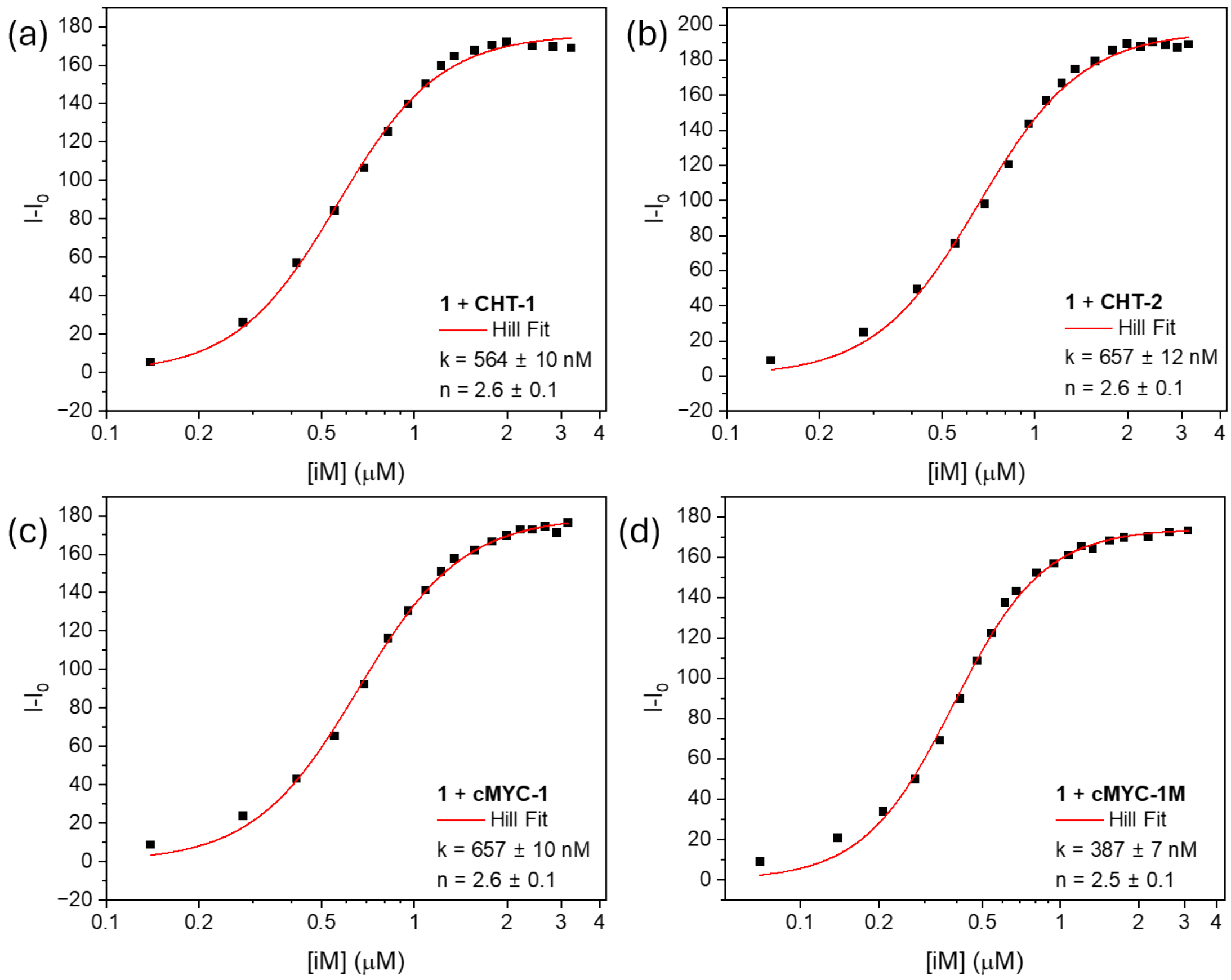

Figure S8. Change in emission intensity of **1** (2.3 μM) with increasing concentration of (a) **CHT-1**, (b) **CHT-2**, (c) **cMYC-1**, and (d) **cMYC-1M** (0 – 3.2 μM) with Hill Fit in 50 mM potassium phosphate buffer, pH 5.5.

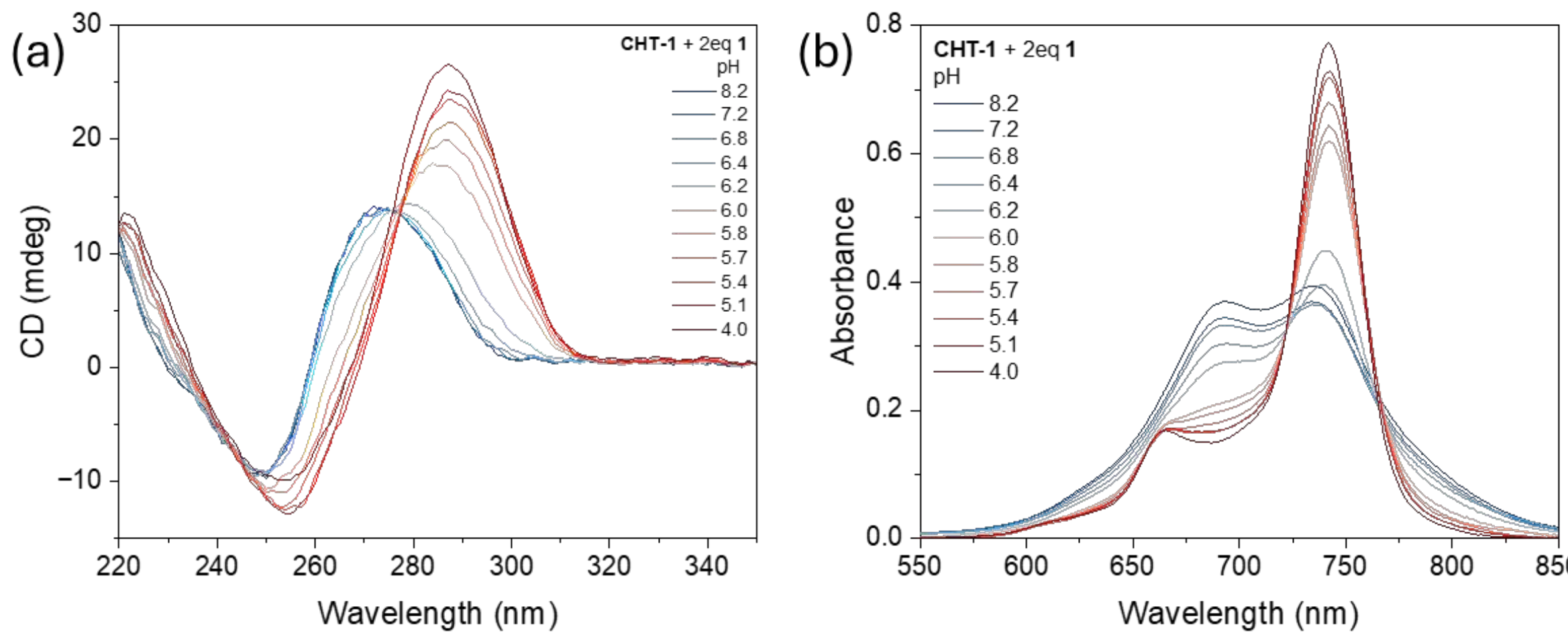

Figure S9. (a) CD spectra of **CHT-1** (3.9 μM) with **1** (7.7 μM) and (b) UV-visible absorption spectra of **1** (7.7 μM) with **CHT-1** (3.9 μM) with decreasing pH in 50 mM potassium phosphate buffer at RT.

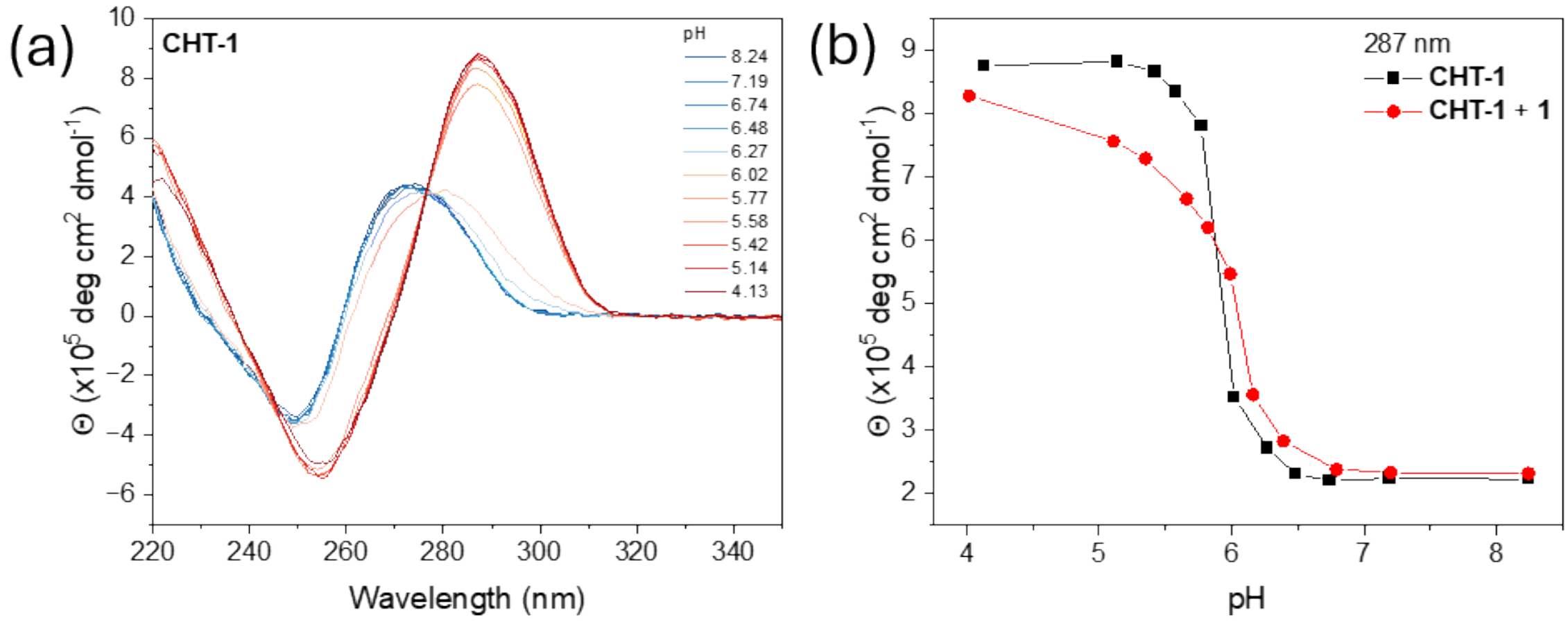

Figure S10. (a) CD spectra of **CHT-1** (3.9 μM) with decreasing pH in 50 mM potassium phosphate buffer. (b) **CHT-1** CD band intensity against pH in presence and absence of **1** (7.7 μM).

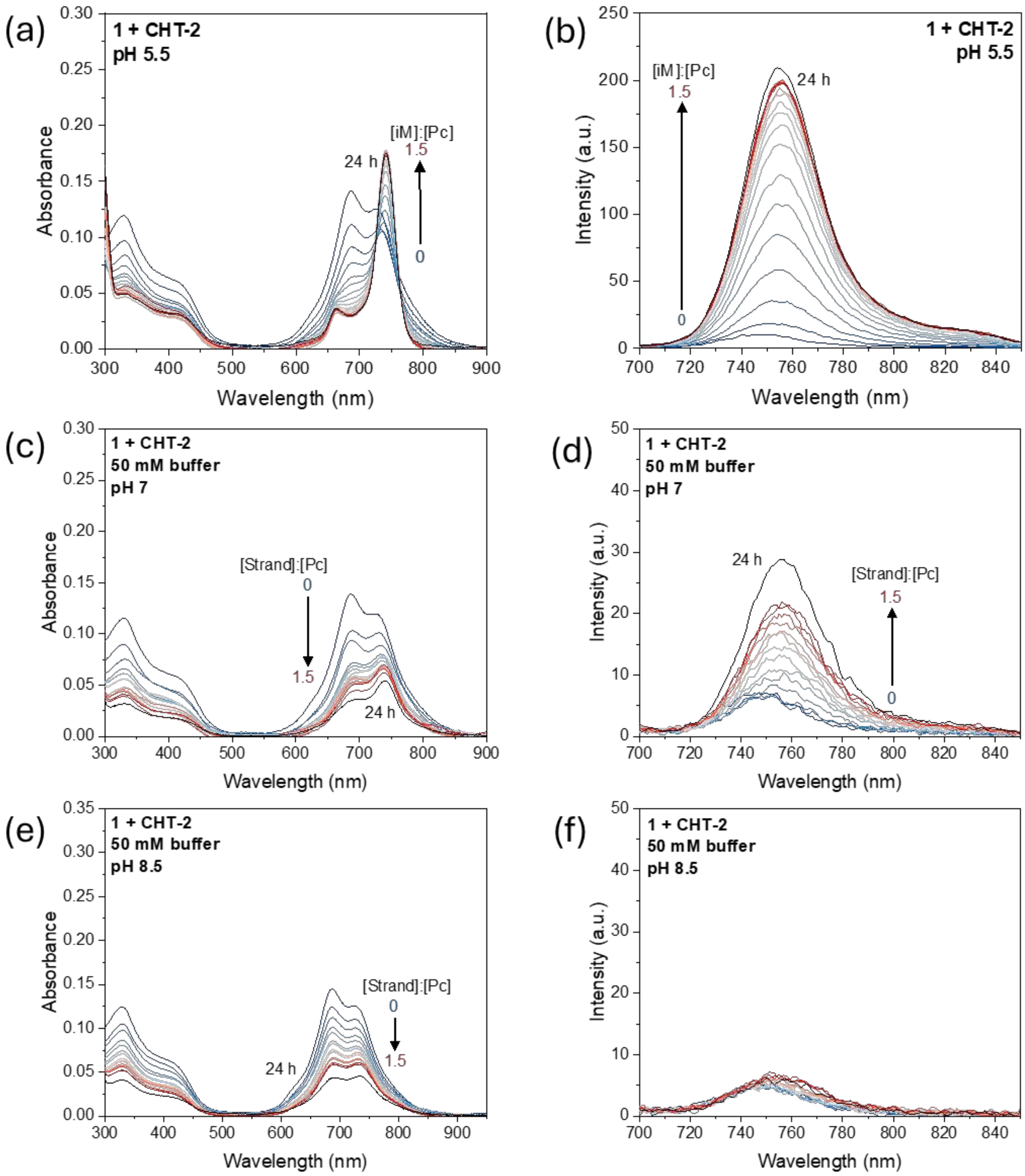

Figure S11. UV-visible absorption (left) and emission (right, λ_{ex} = 650 nm) spectra of **1** (2.3 μM) with increasing concentration of **CHT-2** (0-3.2 μM strand) at pH 5.5 (a, b), pH 7 (c, d) and pH 8.5 (e, f). All spectra were recorded in 50 mM potassium phosphate buffer at RT.

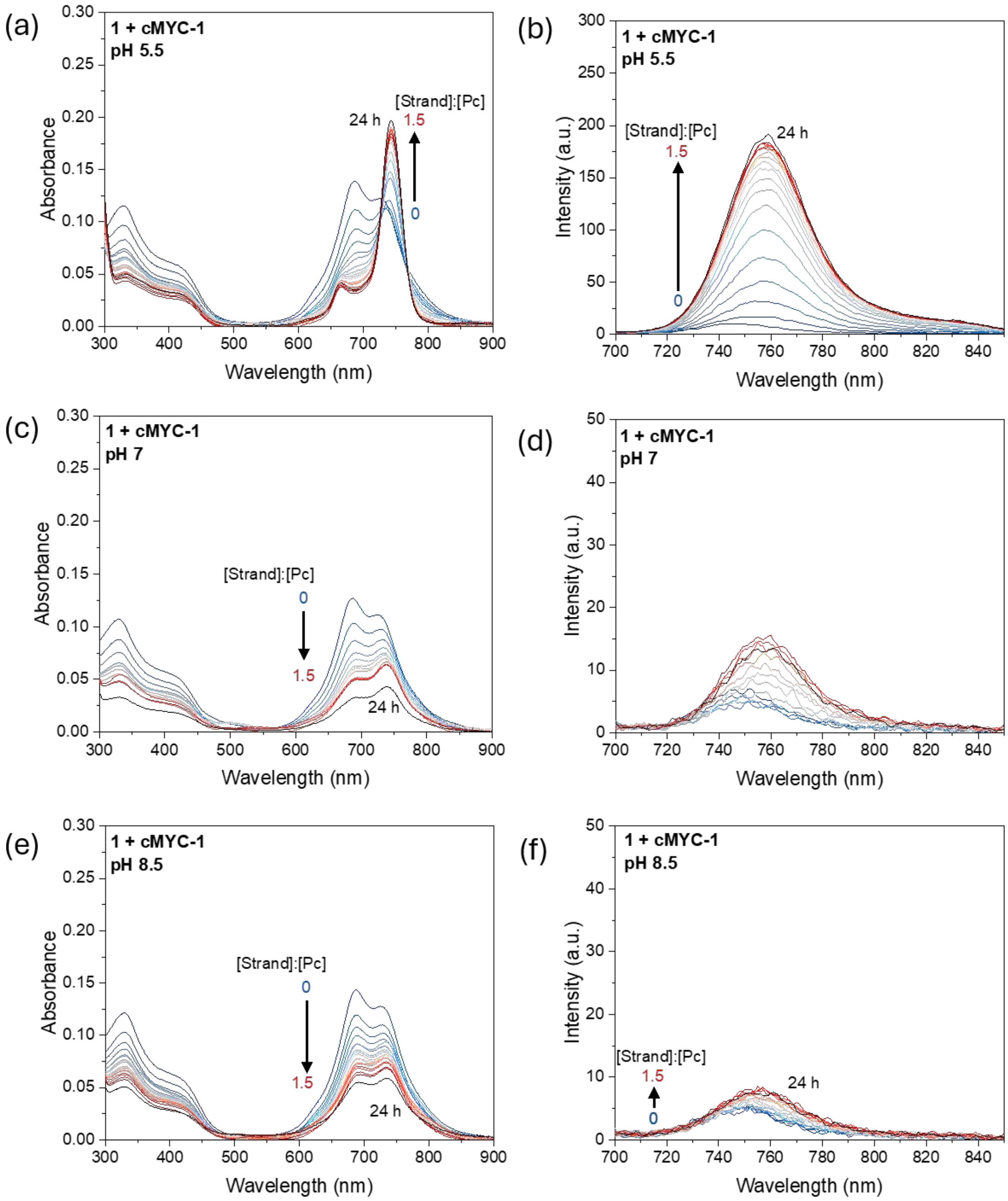

Figure S12. UV-visible absorption (left) and emission (right, λ_{ex} = 650 nm) spectra of **1** (2.3 μM) with increasing concentration of **cMYC-1** (0-3.2 μM strand) at pH 5.5 (a, b), pH 7 (c, d) and pH 8.5 (e, f). All spectra were recorded in 50 mM potassium phosphate buffer at RT.

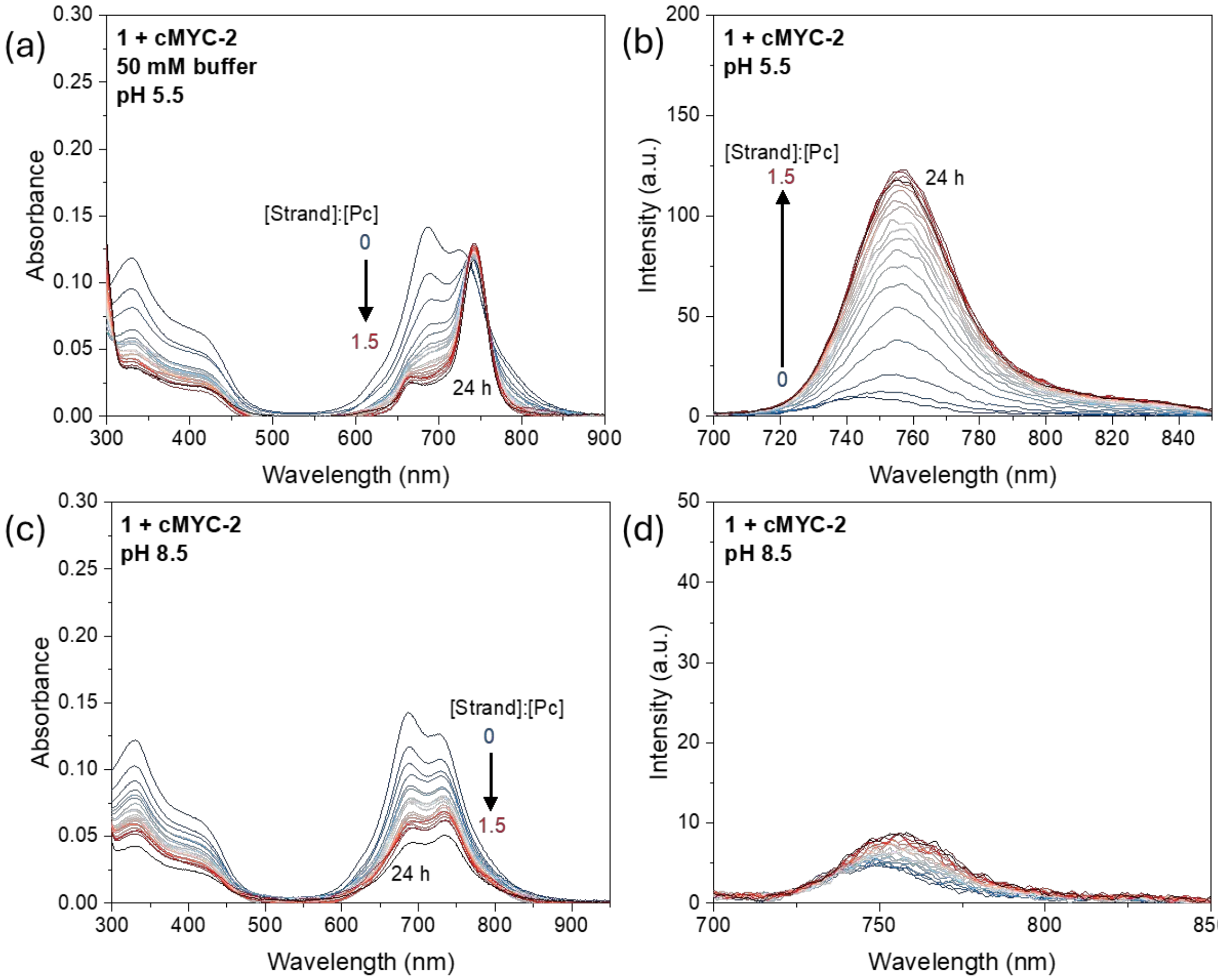

Figure S13. UV-visible absorption (left) and emission (right, λ_{ex} = 650 nm) spectra of **1** (2.3 μM) with increasing concentration of **cMYC-2** (0-3.2 μM strand) at pH 5.5 (a, b) and pH 8.5 (c, d). All spectra were recorded in 50 mM potassium phosphate buffer at RT.

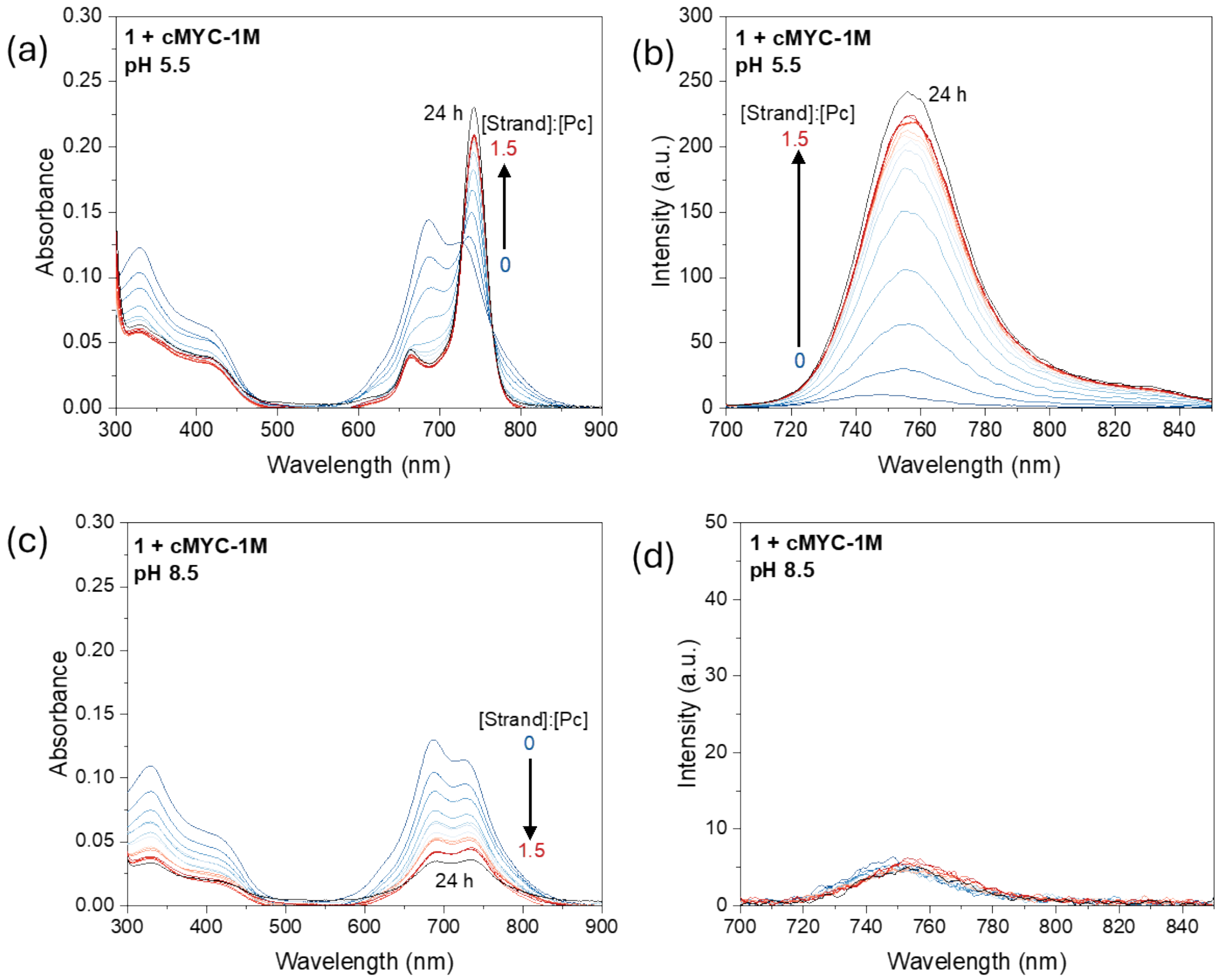

Figure S14. UV-visible absorption (left) and emission (right, λ_{ex} = 650 nm) spectra of **1** (2.3 μM) with increasing concentration of **cMYC-1M** (0-3.2 μM strand) at pH 5.5 (a, b) and pH 8.5 (c, d). All spectra were recorded in 50 mM potassium phosphate buffer at RT.

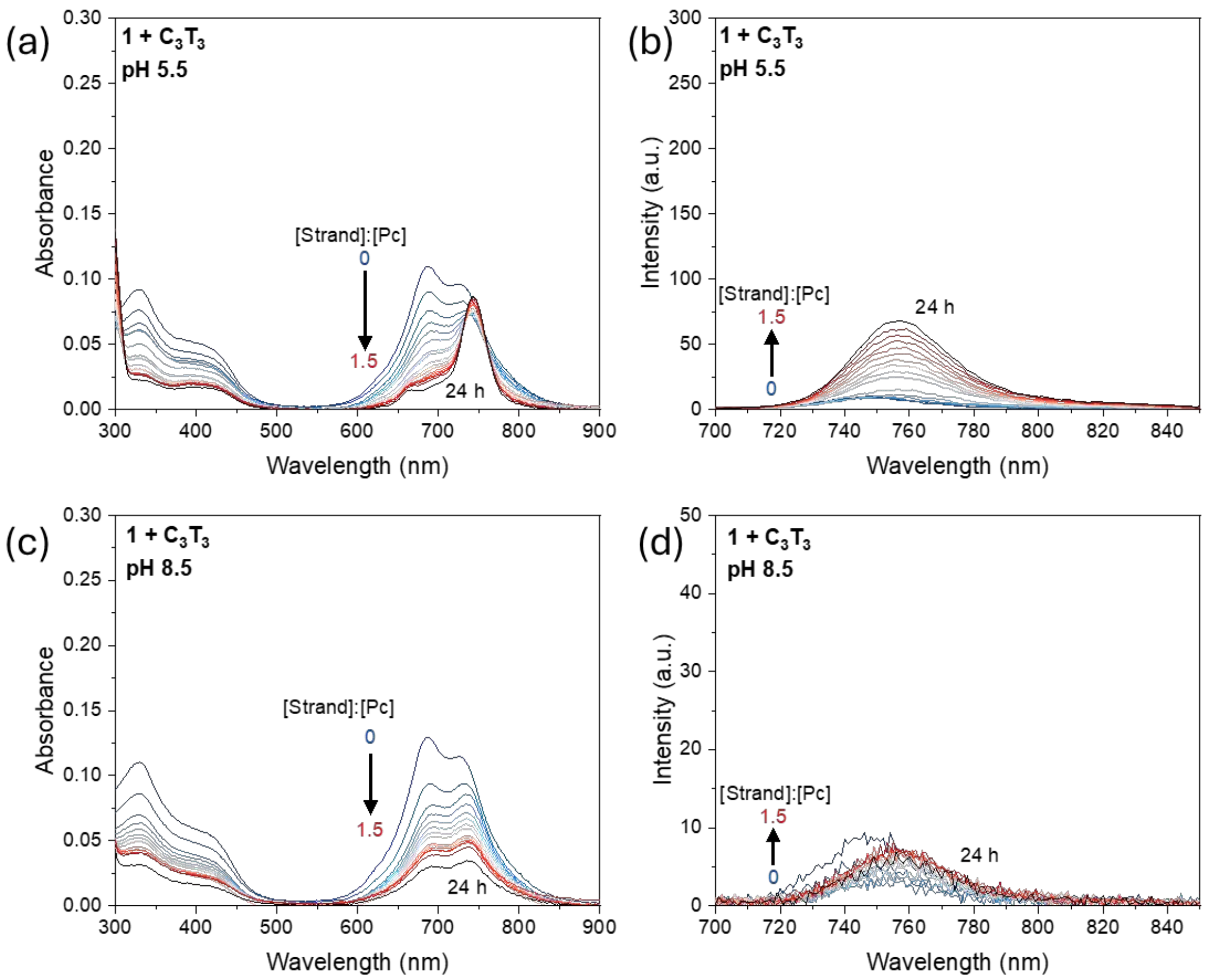

Figure S15. UV-visible absorption (left) and emission (right, λ_{ex} = 650 nm) spectra of **1** (2.3 μM) with increasing concentration of **C_3T_3** (0-3.2 μM strand) at pH 5.5 (a, b) and pH 8.5 (c, d). All spectra were recorded in 50 mM potassium phosphate buffer at RT.

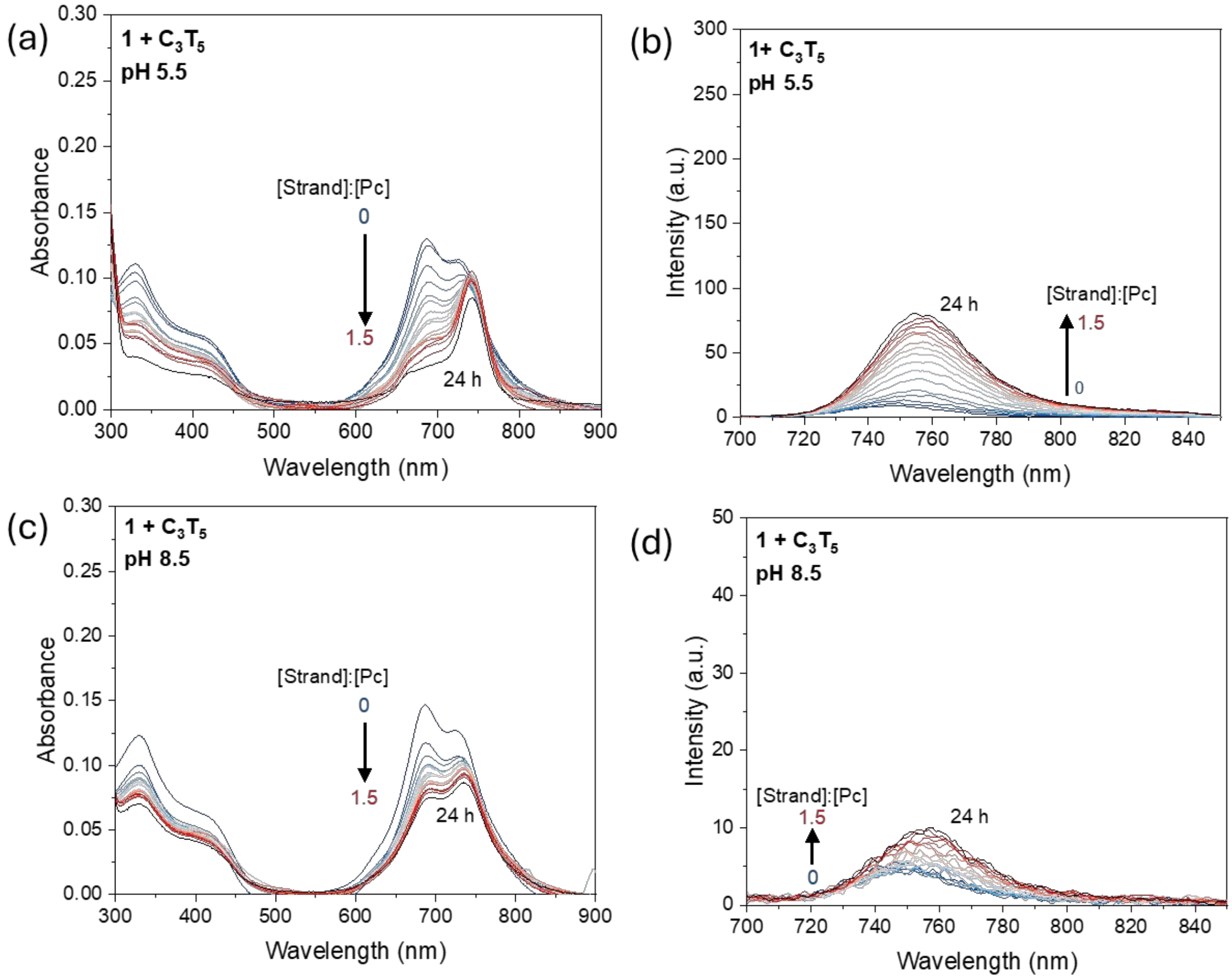

Figure S16. UV-visible absorption (left) and emission (right, λ_{ex} = 650 nm) spectra of **1** (2.3 μM) with increasing concentration of $\mathbf{C_3T_5}$ (0-3.2 μM strand) at pH 5.5 (a, b) and pH 8.5 (c, d). All spectra were recorded in 50 mM potassium phosphate buffer at RT.

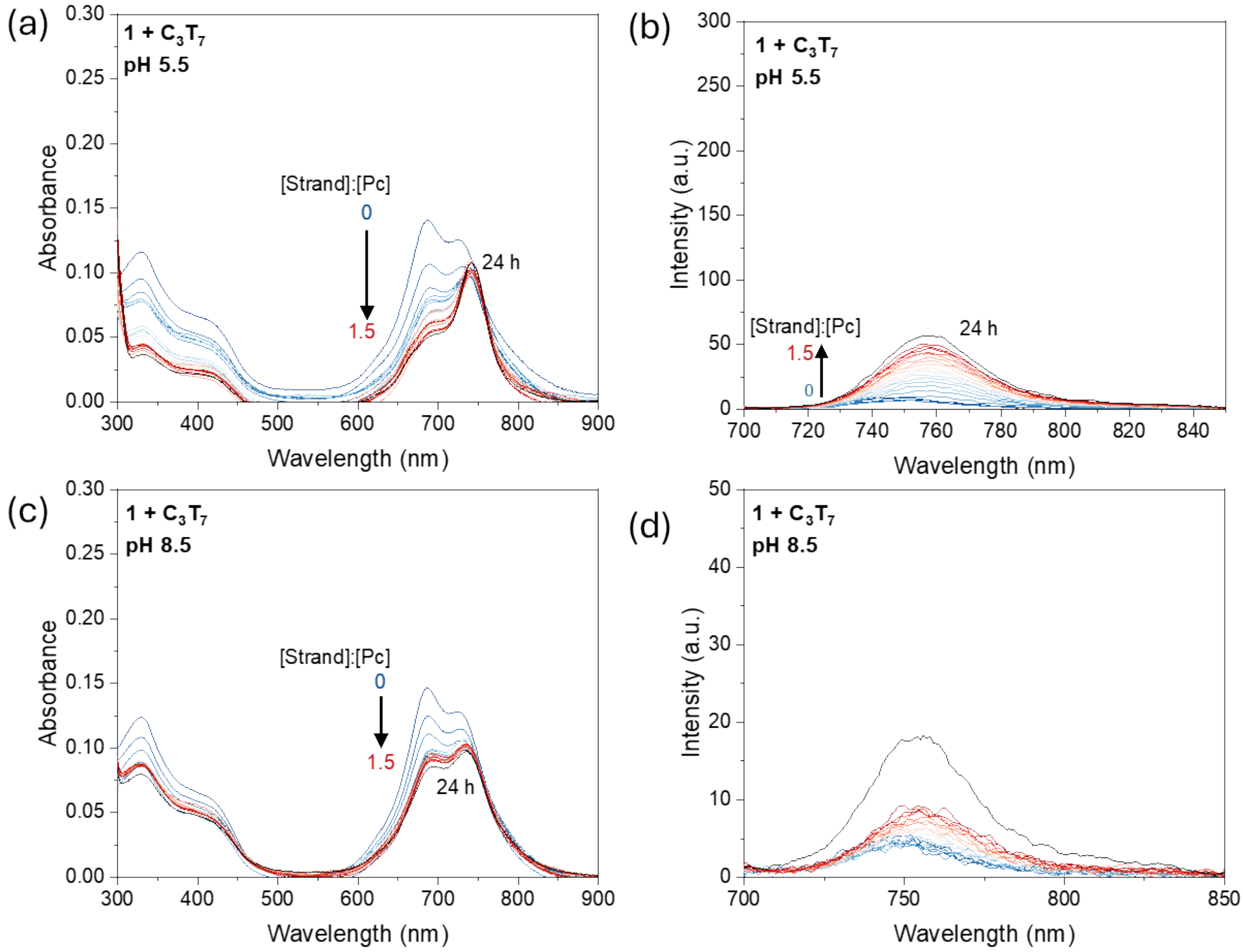

Figure S17. UV-visible absorption (left) and emission (right, λ_{ex} = 650 nm) spectra of **1** (2.3 μM) with increasing concentration of $\mathbf{C_3T_7}$ (0-3.2 μM strand) at pH 5.5 (a, b) and pH 8.5 (c, d). All spectra were recorded in 50 mM potassium phosphate buffer at RT.

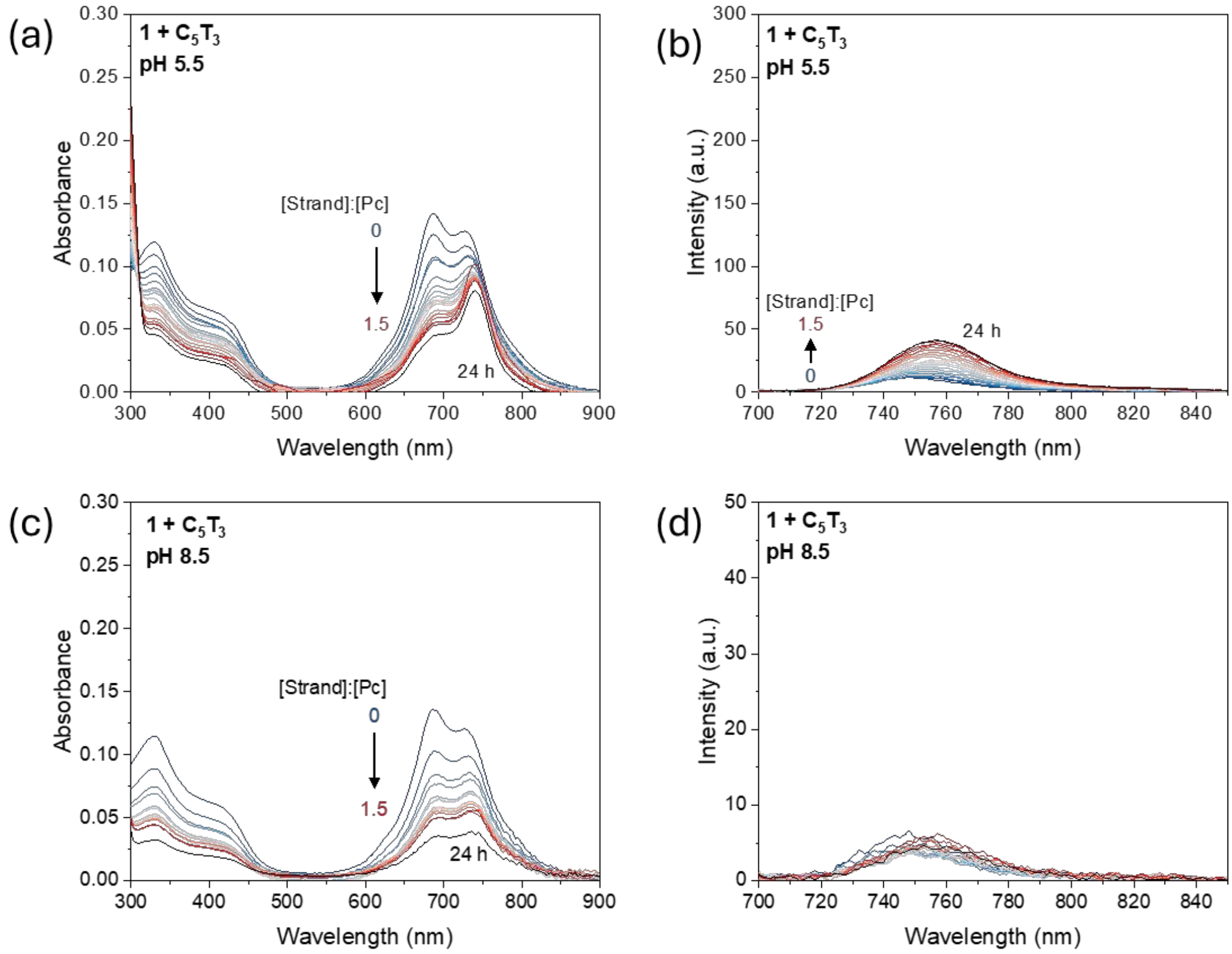

Figure S18. UV-visible absorption (left) and emission (right, λ_{ex} = 650 nm) spectra of **1** (2.3 μM) with increasing concentration of $\mathbf{C_5T_3}$ (0-3.2 μM strand) at pH 5.5 (a, b) and pH 8.5 (c, d). All spectra were recorded in 50 mM potassium phosphate buffer at RT.

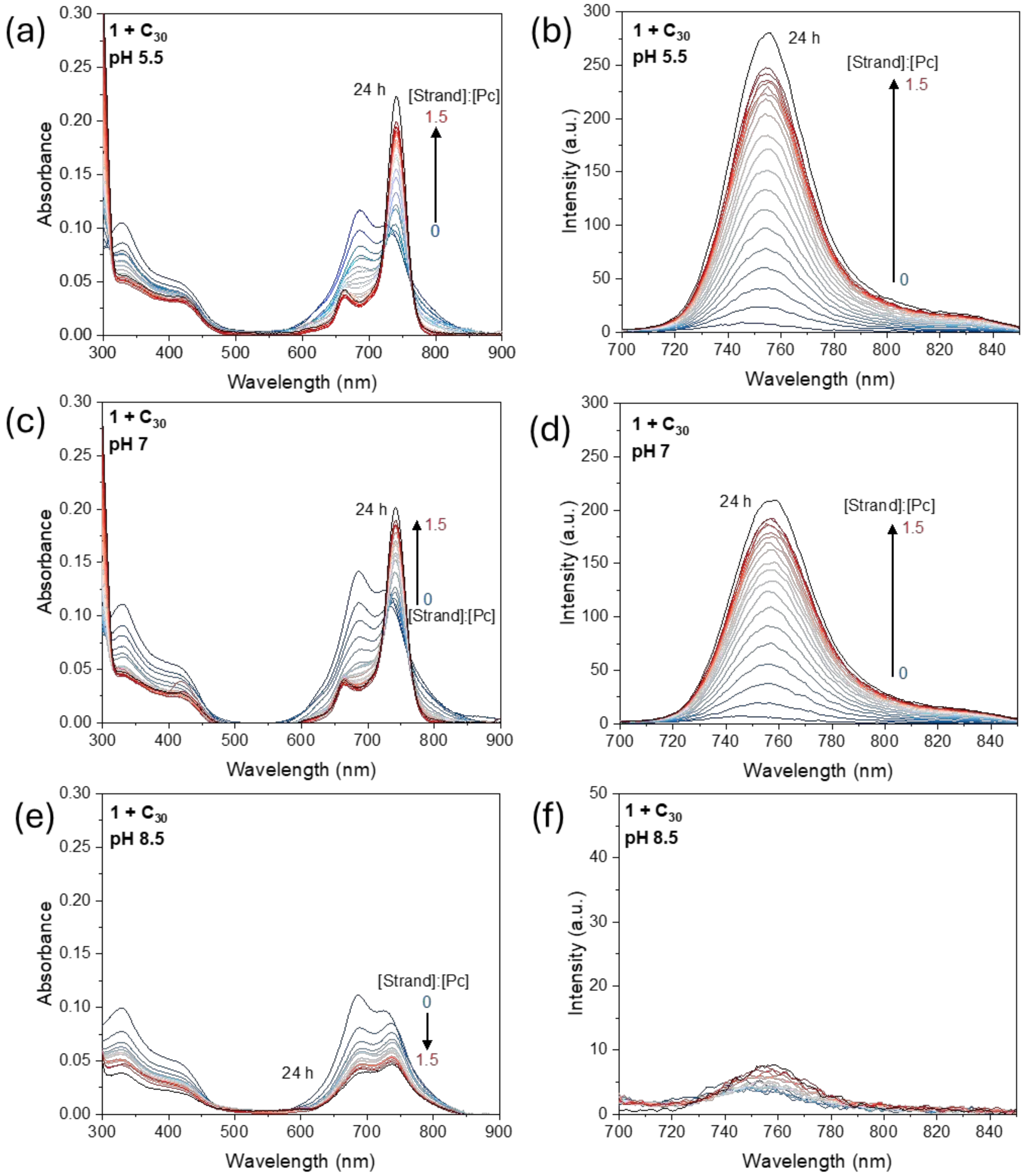

Figure S19. UV-visible absorption (left) and emission (right, λ_{ex} = 650 nm) spectra of **1** (2.3 μM) with increasing concentration of $\mathbf{C_{30}}$ (0-3.2 μM strand) at pH 5.5 (a, b), pH 7 (c, d), and pH 8.5 (e, f). All spectra were recorded in 50 mM potassium phosphate buffer at RT.

Comment on observed emission changes in the presence of iM DNA

In aqueous solution, the small amount of observed emission is due to a small population of emissive monomers, as confirmed by the excitation spectrum matching the known monomer profile in DMSO (Figure S5c). Upon binding to iM DNA, the non-emissive aggregates are disrupted and bind to the DNA as monomers that show fluorescence leading to an overall increase in fluorescence signal. It should be noted that there is a lower quantum yield for **1** in the presence of iM DNA (5%) relative to DMSO (12%). The radiative rate, k_r, for **1** in DMSO solution *vs*. bound to cMYC-1 reduces from 0.062 to 0.026 ns^{-1}, while there is only a modest rise in k_{nr} from 0.456 to 0.481 ns^{-1}. This is consistent with observed hypochromism, because the integral of the absorption band is related to k_r via the Strickler-Berg relation, correlating the reduced absorbance and k_r. It should be noted that there is a potential solvent effect in determining the rates of the different processes.

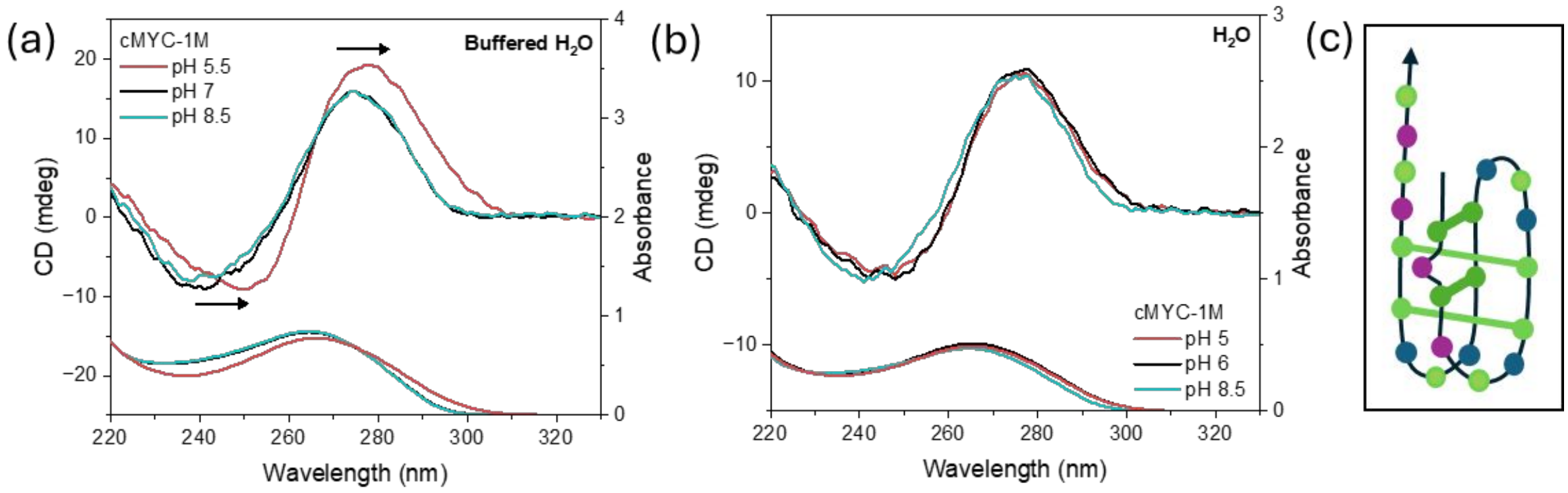

Figure S20. (a) CD (upper data, left axis) and UV-visible absorption (lower data, right axis) spectra of **cMYC-1M** (4.2 μM) at pH 5.5, 7, and 8.5 in 50 mM potassium phosphate buffer at RT. (b) CD (upper data, left axis) and UV-visible absorption (lower data, right axis) spectra of **cMYC-1M** (2.7 μM) at pH 5, 6, and 8.5 in H_2O at RT. (c) Schematic of **cMYC-1M** iM structure in buffered solution at pH 5.5.

Note: The **cMYC-1M** sequence does not form an iM in the absence of buffer (Figure S17b),[15] but absorption and CD spectra characteristic of partial iM formation are observed in buffered solution at pH 5. (Figure S17a). This structure is likely stabilized by the potassium phosphate buffer.[16]

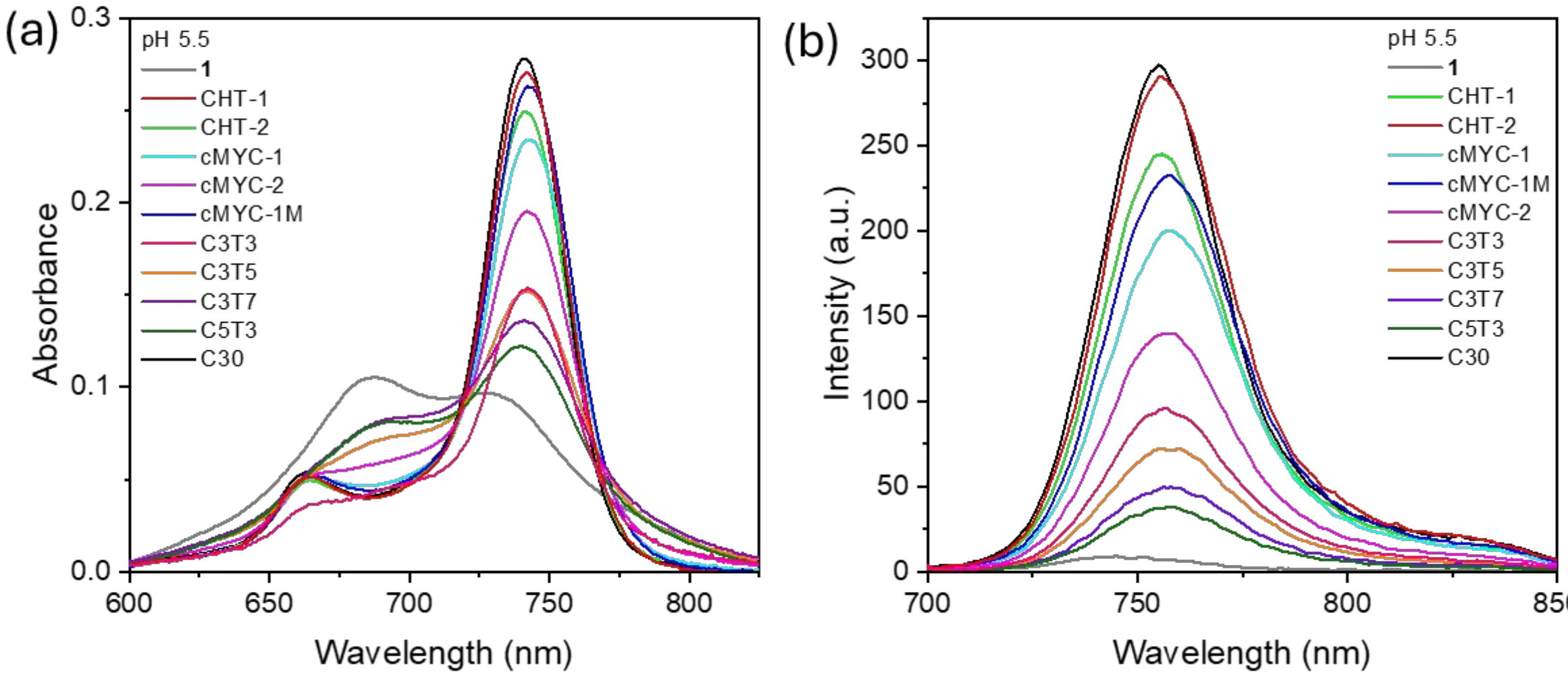

Figure S21. Representative (a) absorption and (b) emission (λ_{ex} = 650 nm) spectra of **1** (2.3 μM) after 24 h incubation with iM-forming sequences (3.5 μM) in 50 mM potassium phosphate buffer (pH 5.5) at RT.

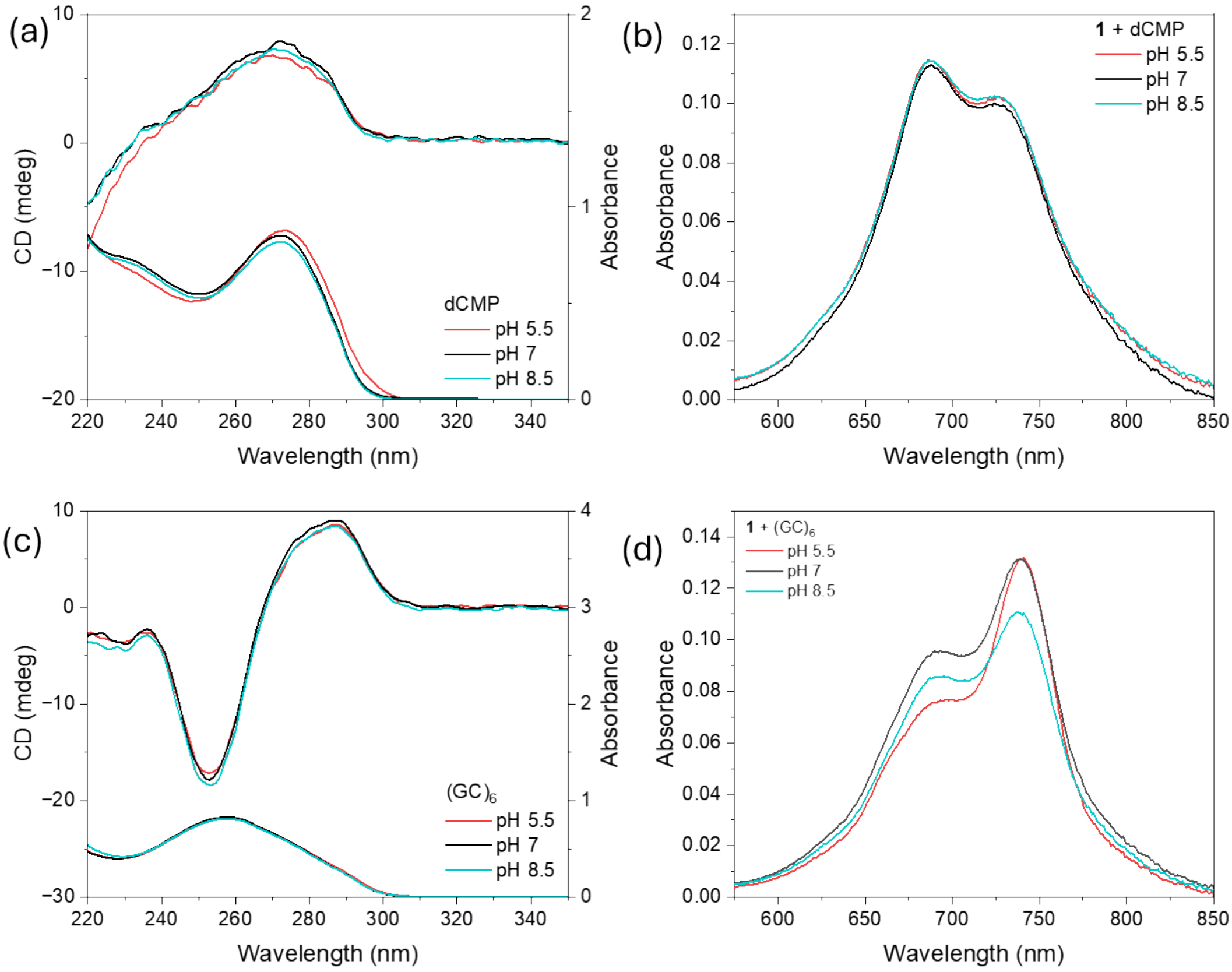

Figure S22. CD (upper data, left axis) and UV-visible absorption (lower data, right axis) of (a) **dCMP** (113 μM Nucleobase) and (c) **(GC)$_6$** (8 μM Strand) at pH 5.5, 7.0, and 8.5. UV-visible absorption spectra of **1** (2.3 μM) with (b) **dCMP** (50 μM Nucleobase) and (d) **(GC)$_6$** (4.6 μM Strand) at pH 5.5, 7.0, and 8.5.

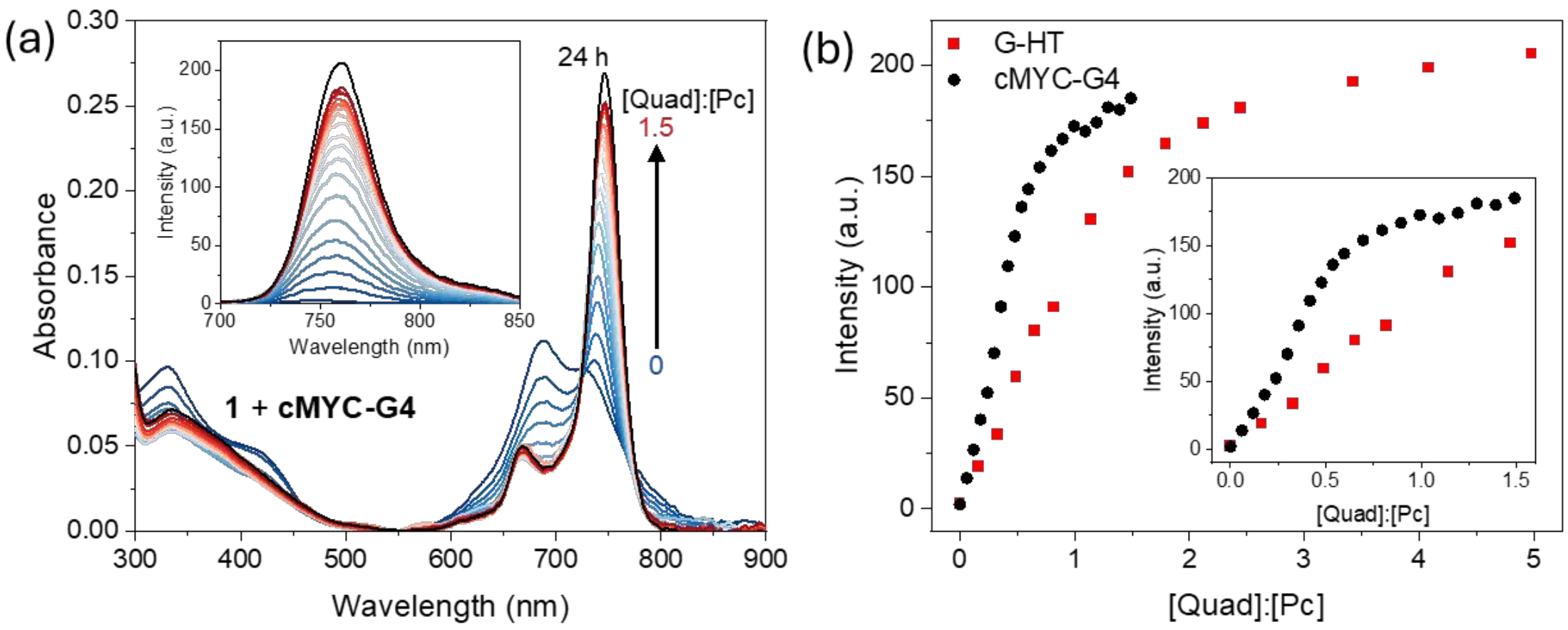

Figure S23. (a) UV-vis absorption and emission (inset, λ_{ex} = 650 nm) spectra of **1** (2.3 μM) titrated against increasing concentration of **cMYC-G4** (0 – 3.2 μM quadruplex) in 50 mM potassium phosphate buffer (pH 7) with 100 mM KCl. (b) Emission intensity of **1** with **G-HT** (757 nm) and **cMYC** (760 nm) against [Quadruplex]:[Pc].

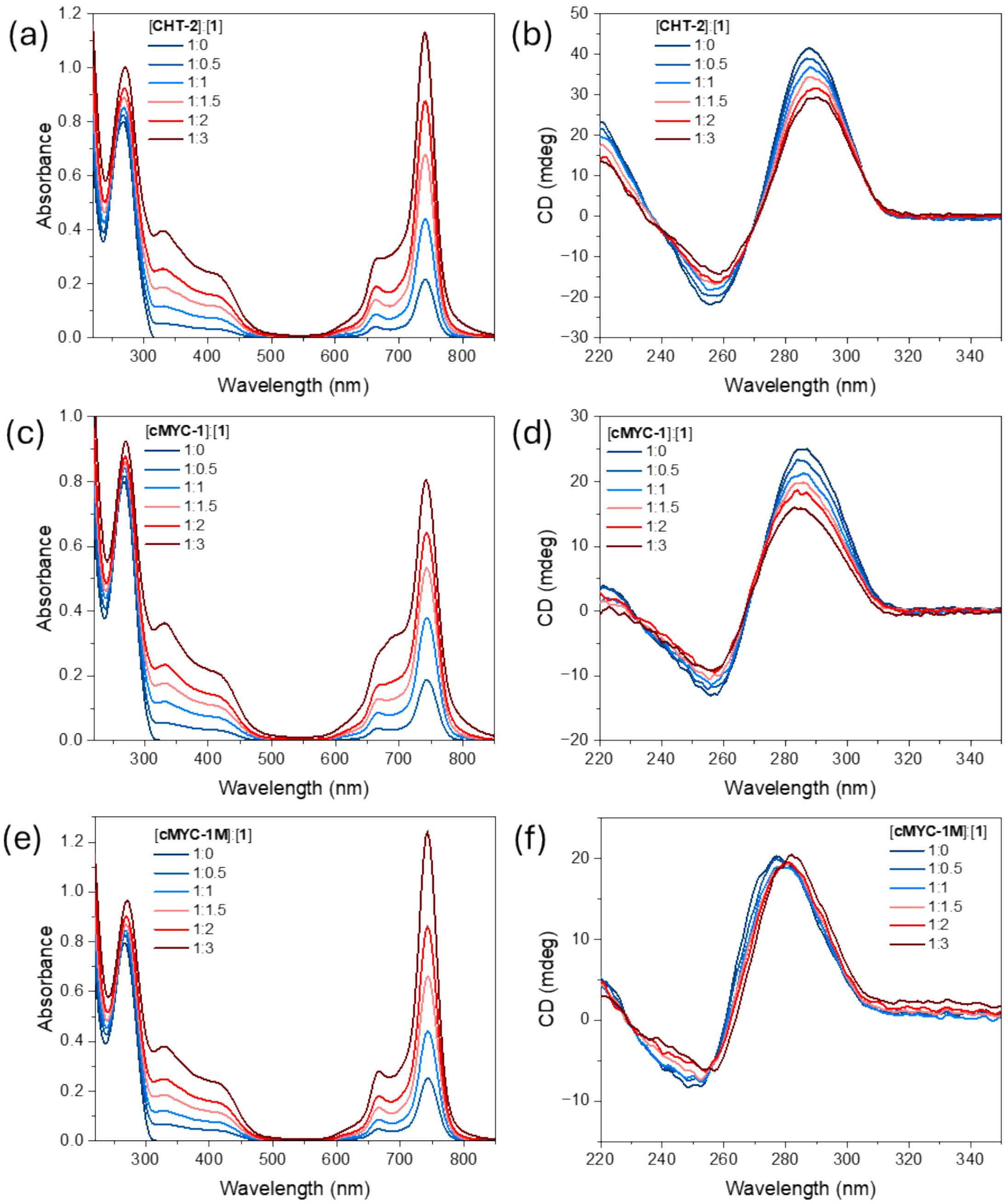

Figure S24. UV-visible absorption (left) and CD (right) spectra of **CHT-2** (a, b) (5 μM), **cMYC-1** (c, d) (4.8 μM), **cMYC-1M** (e, f) (4.4 μM) with addition of **1** (0 – 15 μM). All spectra were recorded in 50 mM potassium phosphate buffer (pH 5.5) at RT.

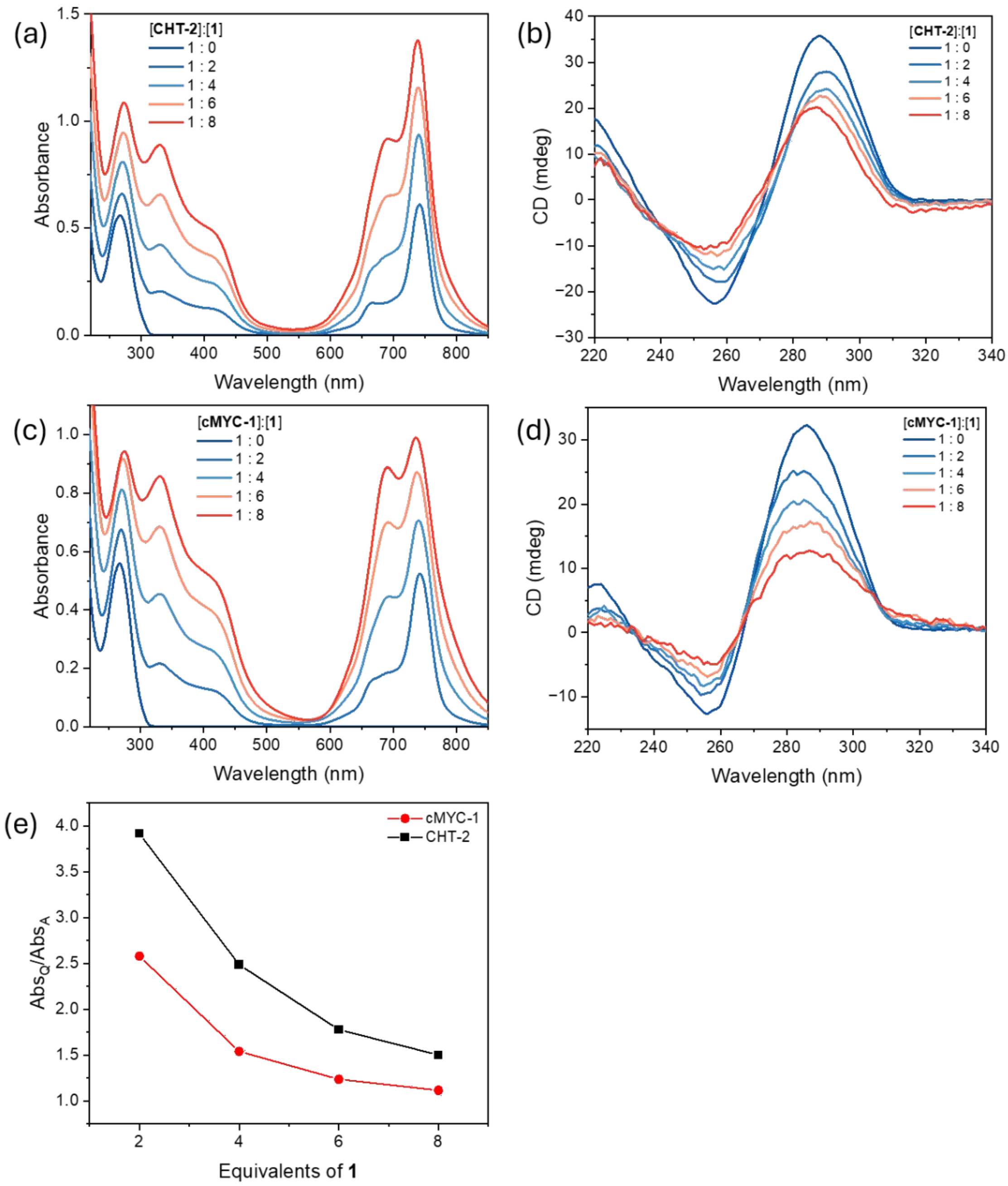

Figure S25. UV-visible absorption (left) and CD (right) spectra of **CHT-2** (a, b) (3.2 μM) and **cMYC-1** (c, d) (3 μM) with 0 to 8 molar equivalents of **1** (0 – 25 μM) in 50 mM potassium phosphate buffer, pH 5.5 at RT. (e) Comparison of ratio of Q-band (~740 nm) and aggregate band (~687 nm) absorbance for increasing equivalents of **1** with **CHT-2** and **cMYC-1**.

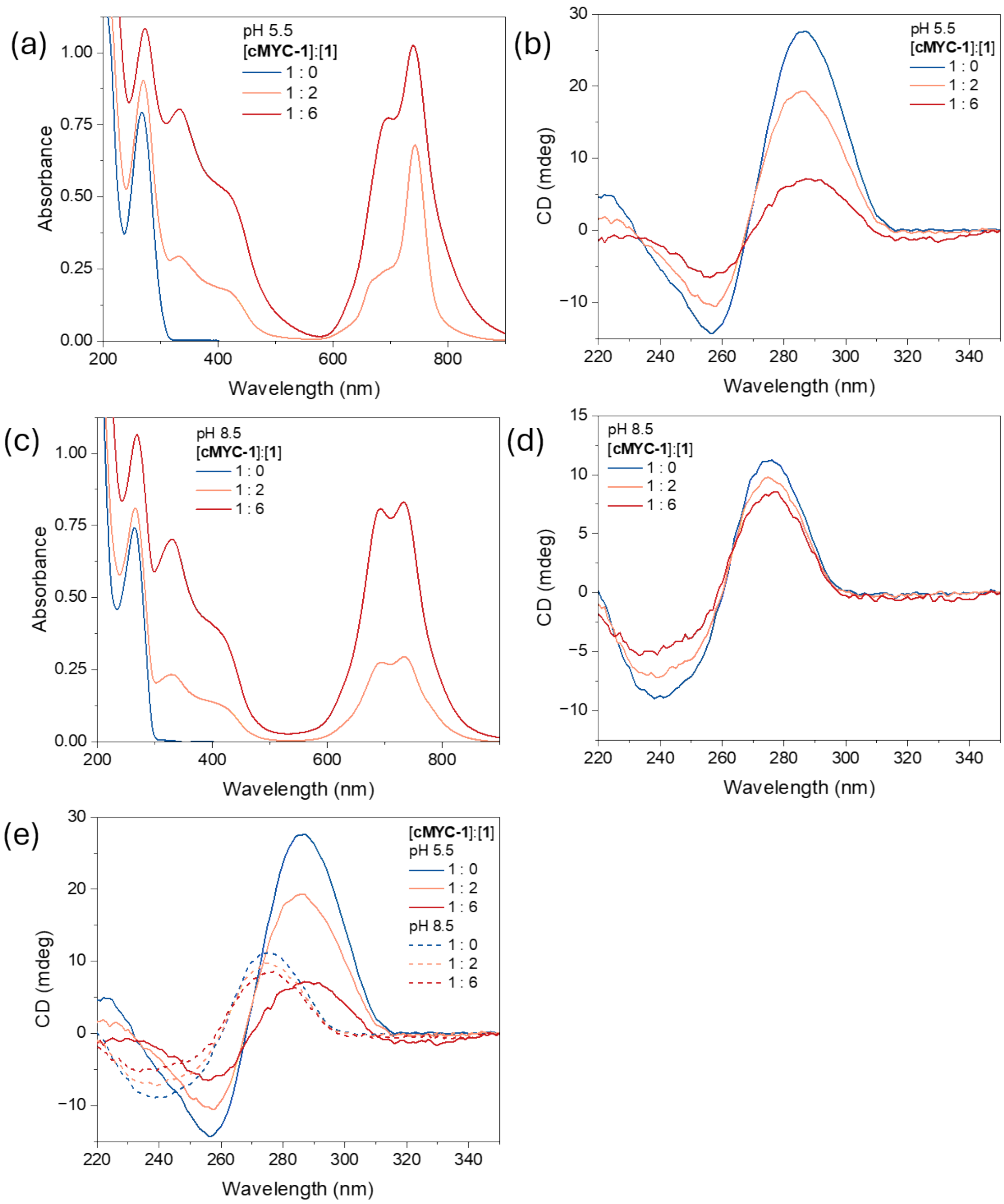

Figure S26. UV-visible absorption and CD spectra of **cMYC-1** (4.8 μM) with increasing concentration of **1** (0 – 29 μM) in 50 mM potassium phosphate buffer at (a, c) pH 5.5 and (b, d) pH 8.5. (e) Comparison of CD spectra at pH 5.5 and pH 8.5.

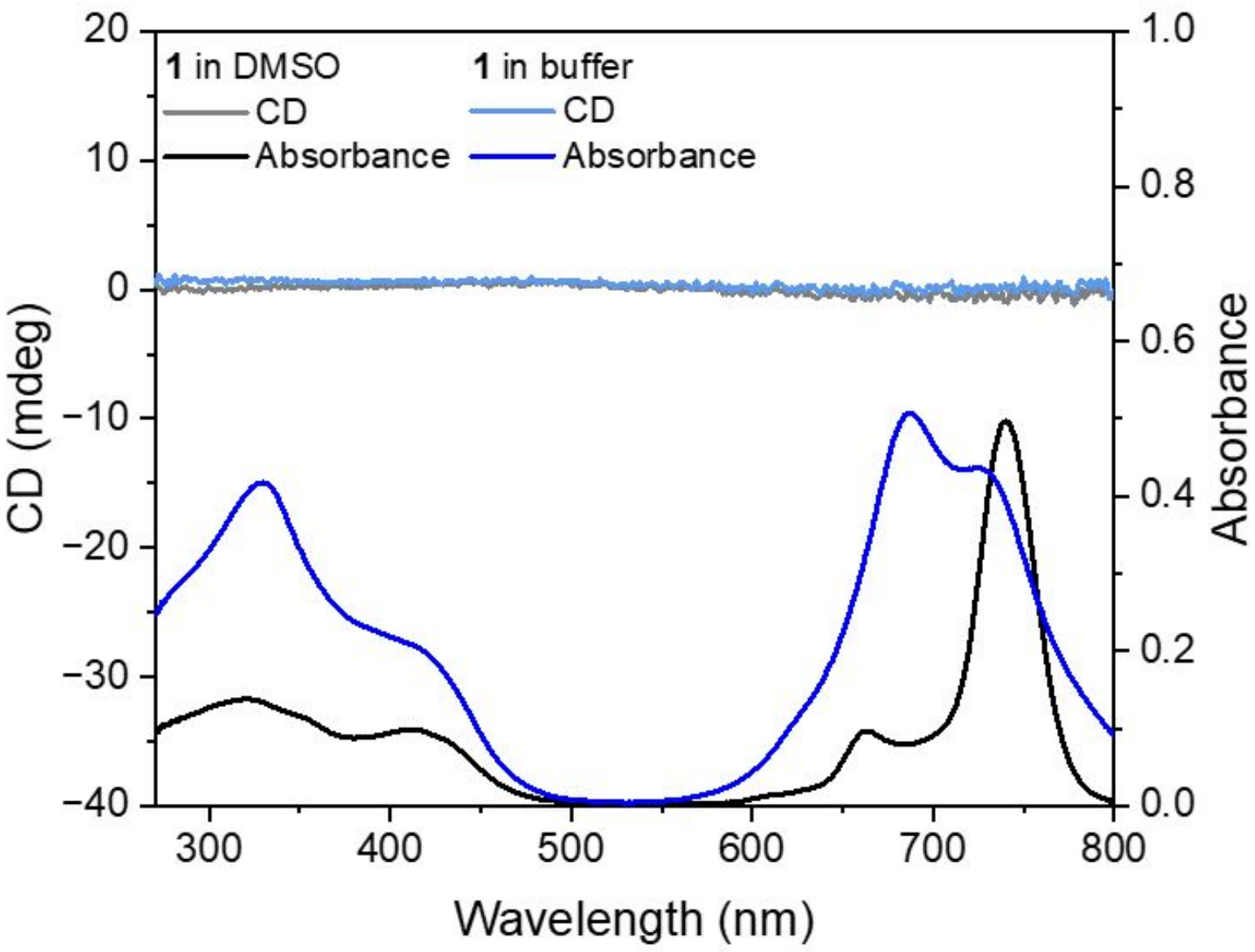

Figure S27. CD (upper data, left axis) and UV-visible absorption (lower data, right axis) spectra of **1** (29 µM) in DMSO and **1** (145 µM) in 50 mM potassium phosphate buffer, pH 5.5 recorded in a 1 mm pathlength cuvette.

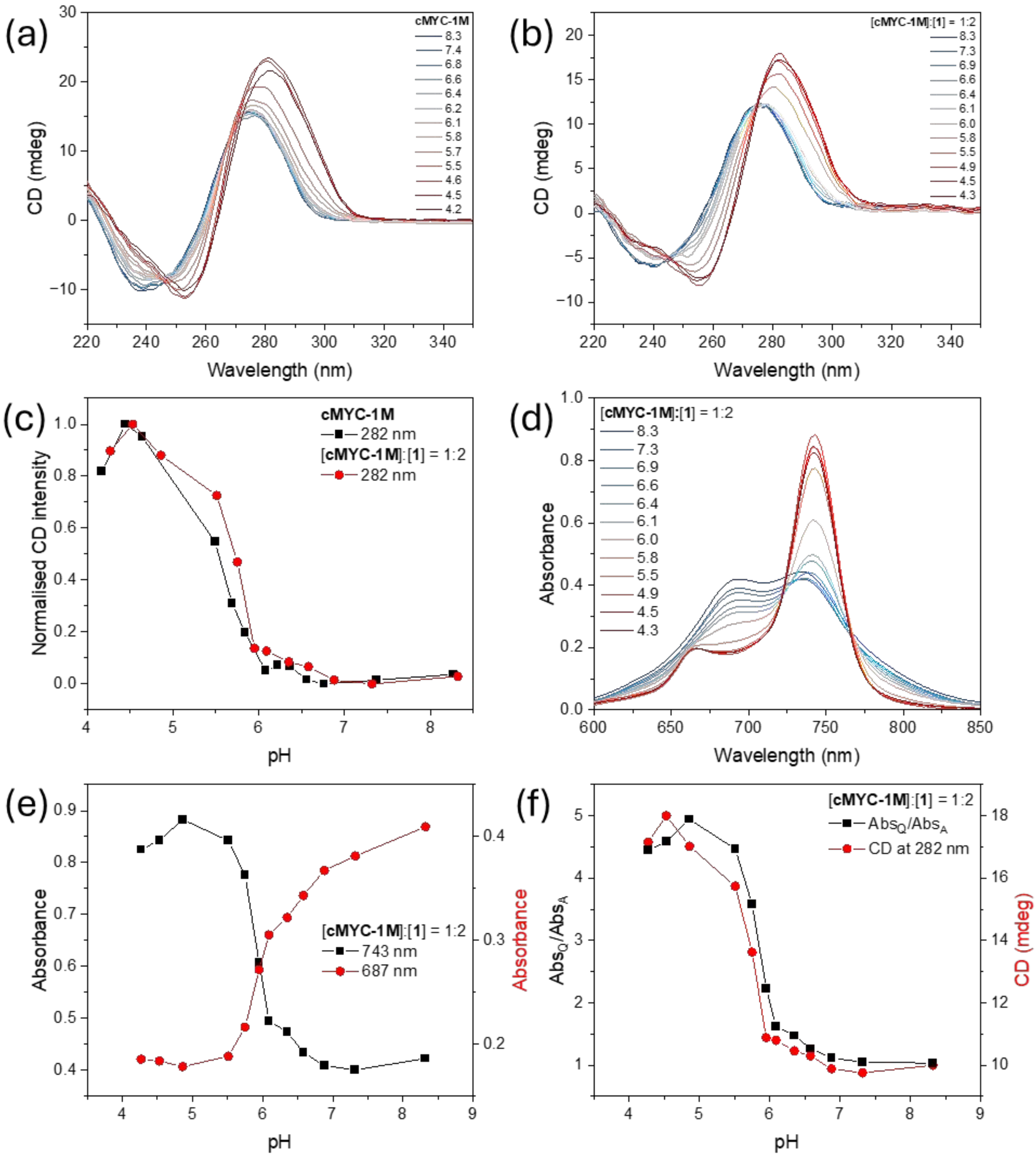

Figure S28. CD spectra of **cMYC-1M** (4.3 μM) in (a) absence and (b) presence of **1** (8.3 μM) with decreasing pH. (c) Normalized **cMYC-1M** CD band intensity against pH. (d) UV-visible absorption spectra of **1** (8.3 μM) with **cMYC-1M** (4.3 μM) with decreasing pH. (e) Absorbance of **1** at 743 nm (left axis) and 687 nm (right axis) as function of pH. (f) Ratio of absorbance at 743 nm and 687 nm of **1** (left axis) and CD band intensity of **cMYC-1M** (right axis) as function of pH. All spectra were recorded in 50 mM potassium phosphate buffer at RT.

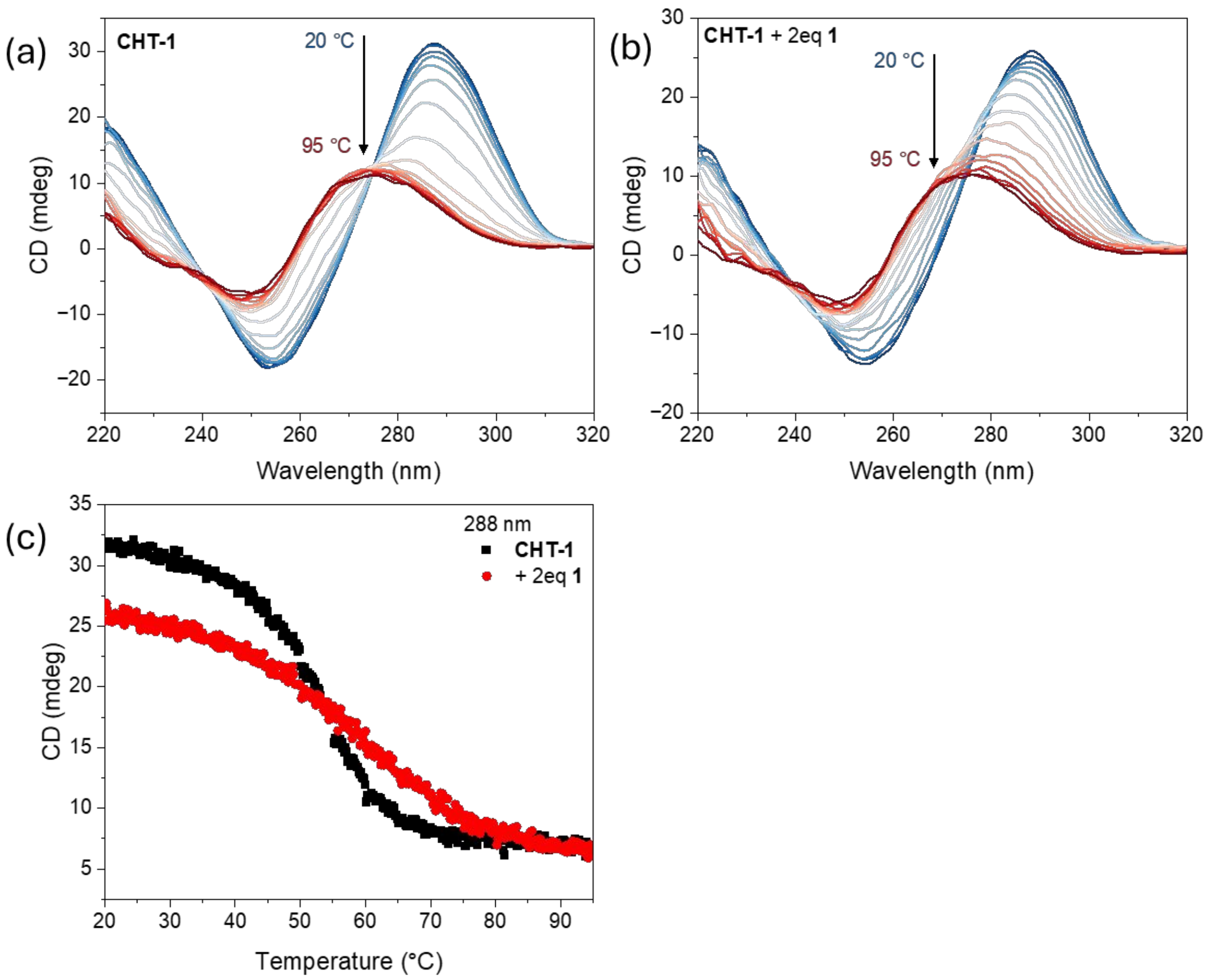

Figure S29. (a, b) CD spectra at 5 °C increments between 20 °C and 95 °C of (a) **CHT-1** (4.2 μM) and (b) with addition of **1** (8.4 μM). (c) CD band intensity of **CHT-1** at 288 nm in presence and absence of **1** as function of temperature. All spectra were recorded in 20 mM sodium acetate buffer (pH 5.5) with heating rate of 1 °/min.

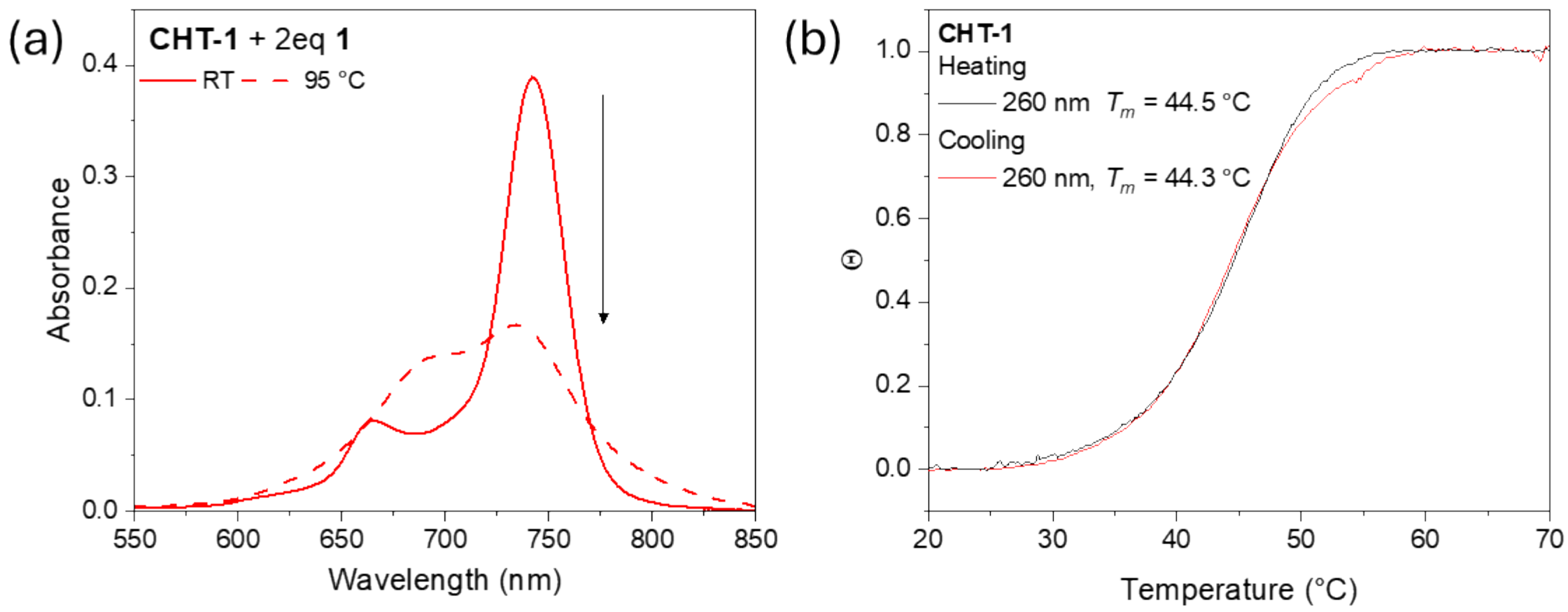

Figure S30. (a) UV-visible absorption spectrum of **1** (3.6 μM) with **CHT-1** (1.8 μM) at RT and 95 °C. (b) Fraction of unfolded **CHT-1** (1.8 μM) calculated at 260 nm as function of temperature when heated at rate of 1 °C/min and cooled at rate of 1 °C/min. All spectra were recorded in 20 mM sodium acetate buffer (pH 5.5).

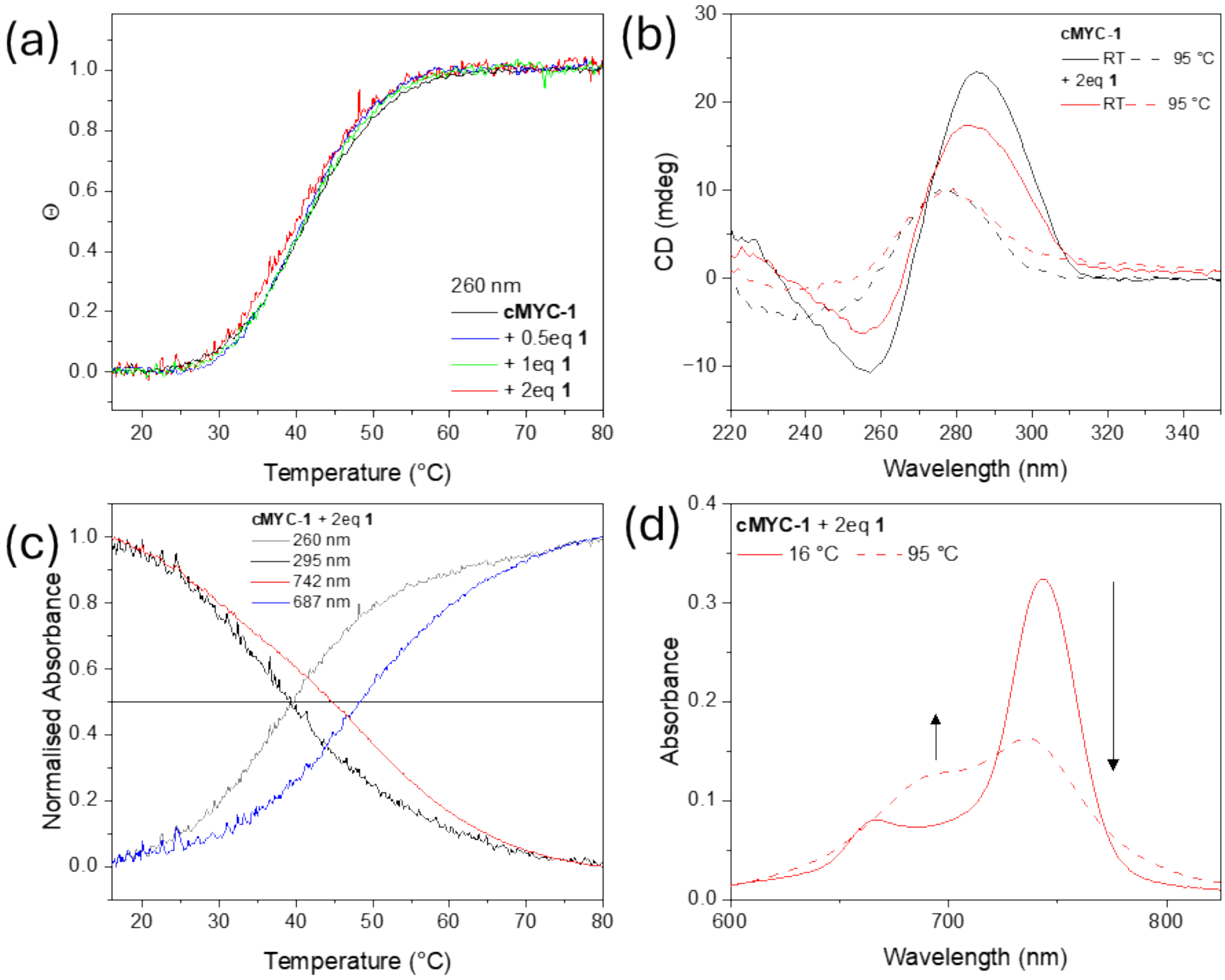

Figure S31. (a) Fraction of unfolded **cMYC-1** (2.0 µM) as function of temperature with **1** (0 – 4.0 µM). (b) CD spectra of **cMYC-1** (4.0 µM) with **1** (8.0 µM) at RT and 95 °C. (c) Normalised absorbance bands of **cMYC-1** (2.0 µM) with **1** (4.0 µM). (d) UV-visible absorption spectrum of **1** (4.0 µM) with **cMYC-1** (2.0 µM) at 16 °C and 95 °C. All spectra were recorded in 20 mM sodium acetate buffer (pH 5.5) with heating rate of 1 °C/min.

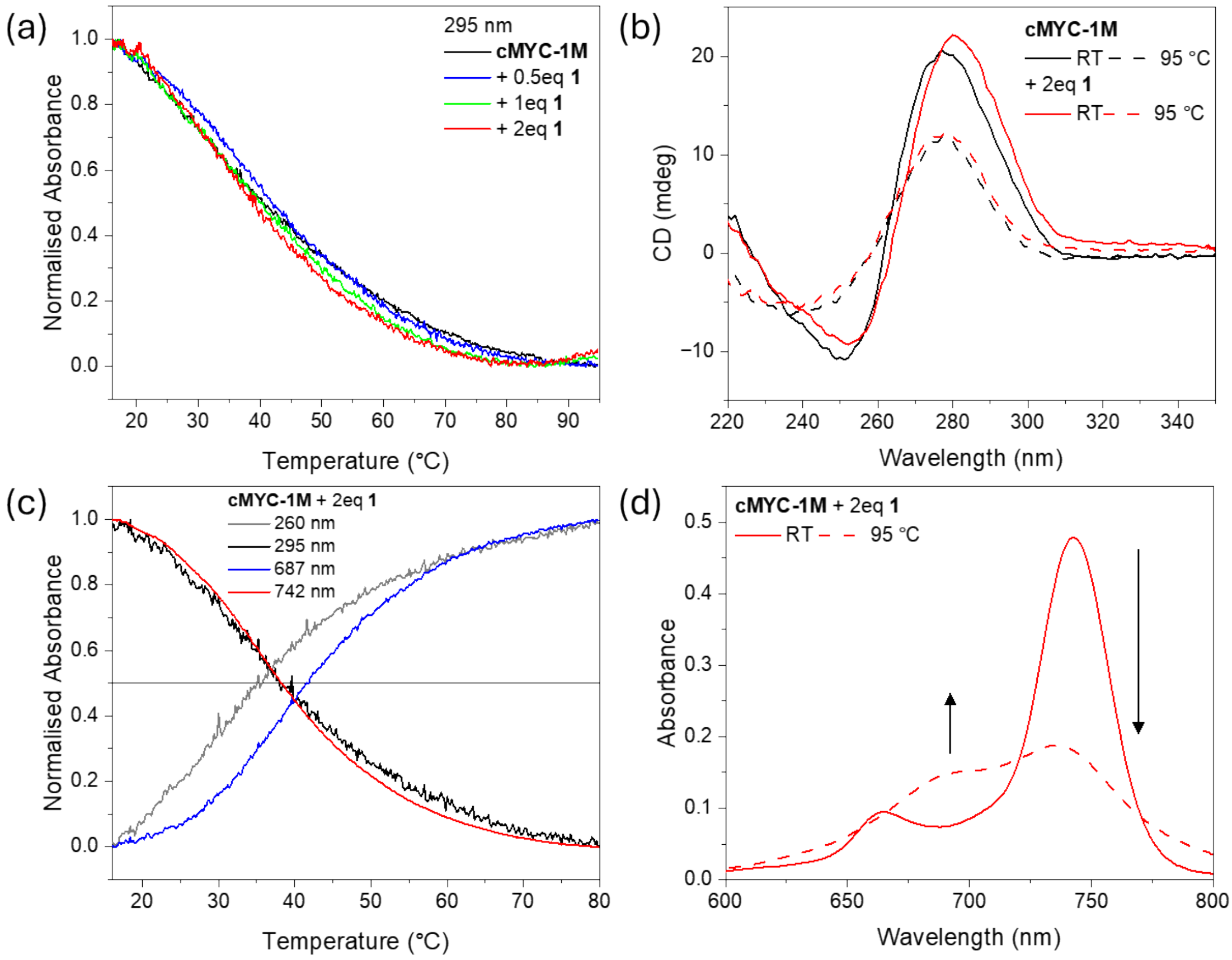

Figure S32. (a) Normalized absorbance of **cMYC-1M** (2.1 µM) at 295 nm with **1** (0 – 4.2 µM) as function of temperature. (b) CD spectra of **cMYC-1M** (4.4 µM) with **1** (8.8 µM) at RT and 95 °C. (c) Normalized absorbance bands of **cMYC-1** (2.1 µM) with **1** (4.2 µM) as a function of temperature. (d) UV-visible absorption spectra of **1** (4.2 µM) with **cMYC-1** (2.1 µM) at RT °C and 95 °C. All spectra were recorded in 20 mM sodium acetate buffer (pH 5.5) with heating rate of 1 °C/min.

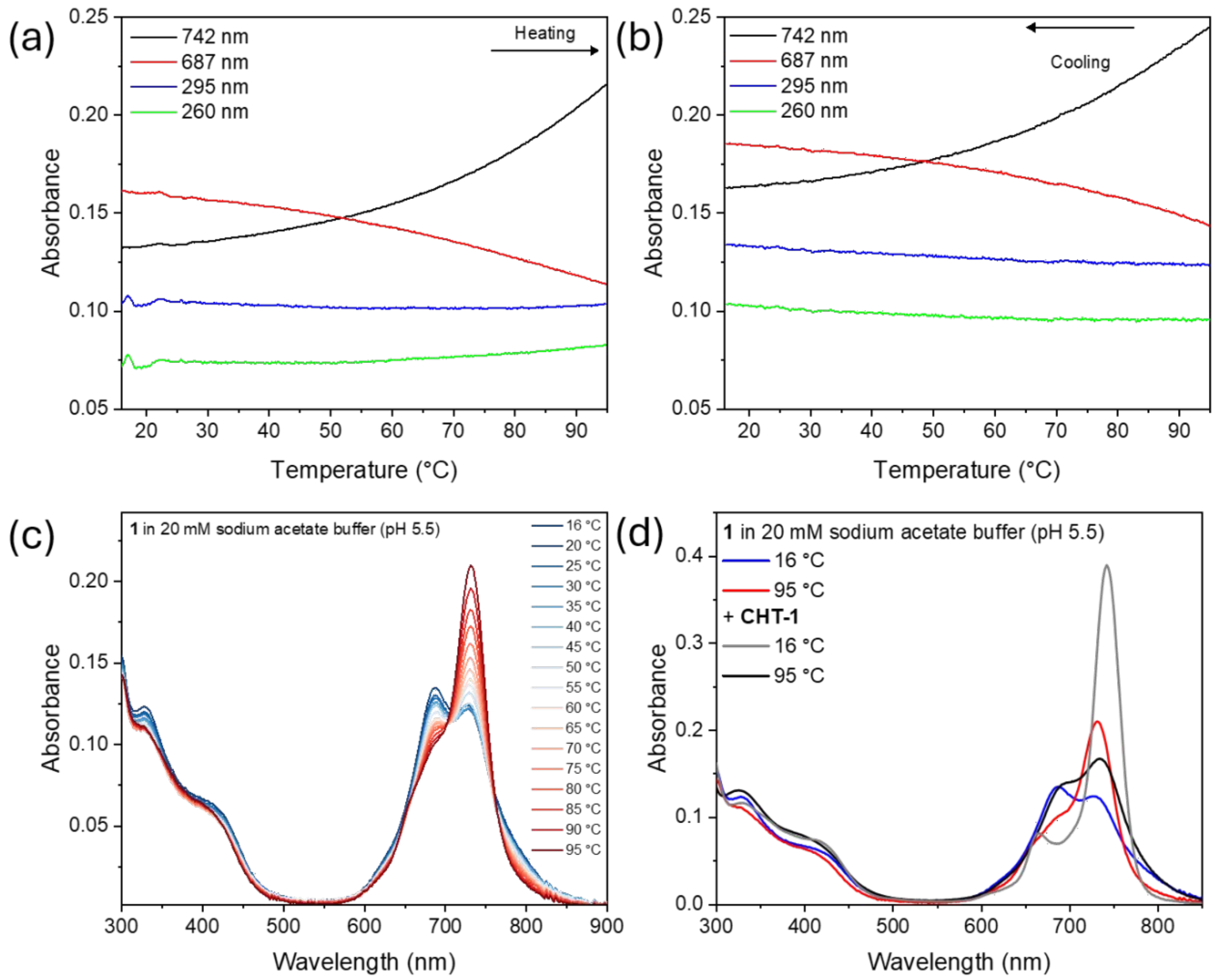

Figure S33. Absorbance at 742 nm, 687 nm, 295 nm, and 260 nm of solution of **1** (3.6 μM) as a function of temperature when (a) heated and (b) cooled between 16 and 95 °C. (c) UV-visible absorption spectra of **1** (3.6 μM) when heated between 16 and 95 °C. (d) UV-visible absorption spectra of **1** (3.6 μM) in buffer and in presence of CHT-2 (1.8 μM) at 16 and 95 °C. All spectra were recorded in 20 mM sodium acetate buffer (pH 5.5) with heating/cooling rate of 1 °C/min.

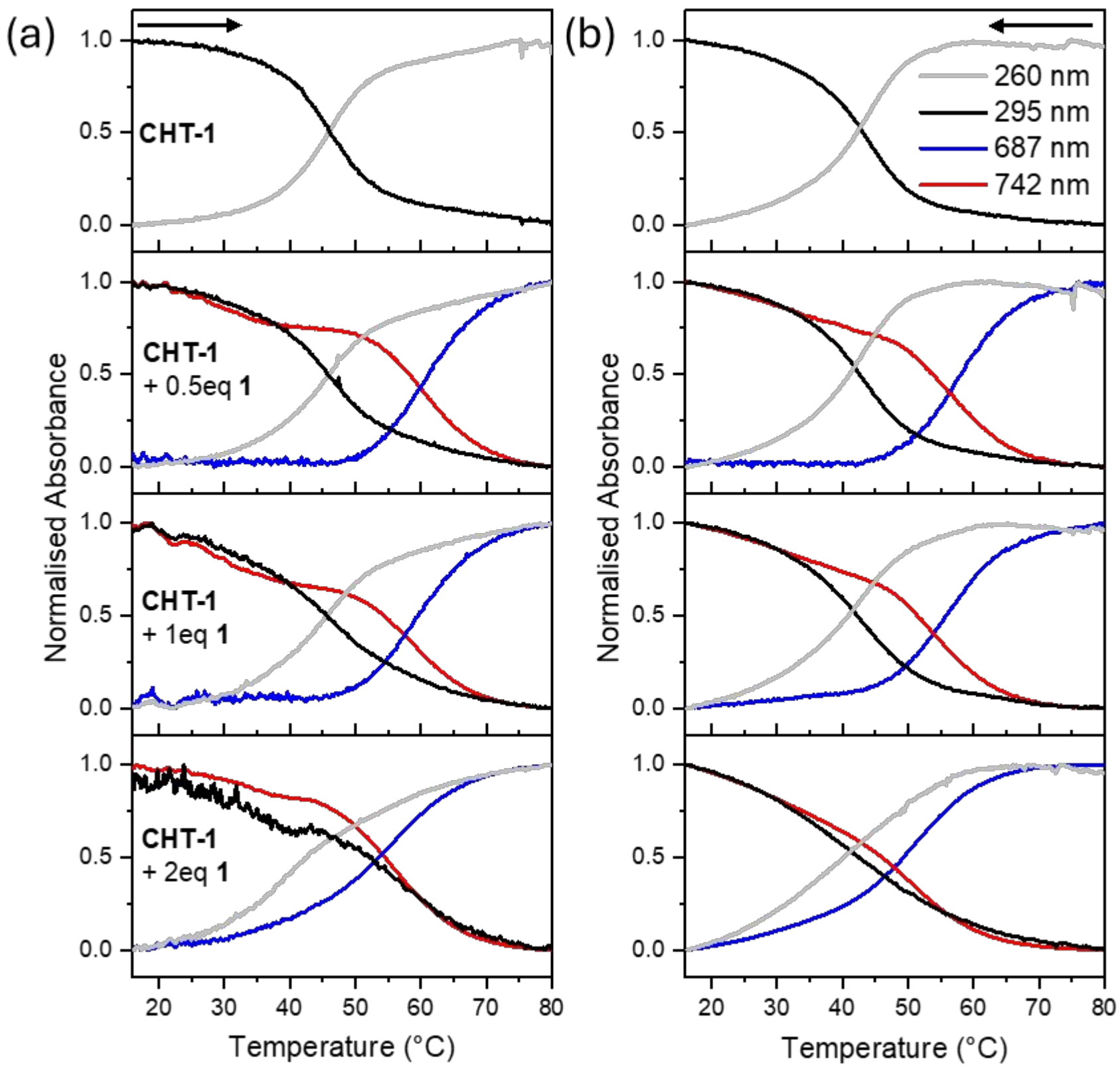

Figure S34. Normalized absorbance of **CHT-1** (1.8 μM) at 260 nm and 295 nm and **1** (0 – 3.6 μM) at 687 nm and 742 nm as a function of temperature when (c) heated and (d) cooled with ramp rate of 1 °C/min in 20 mM sodium acetate buffer (pH 5.5).

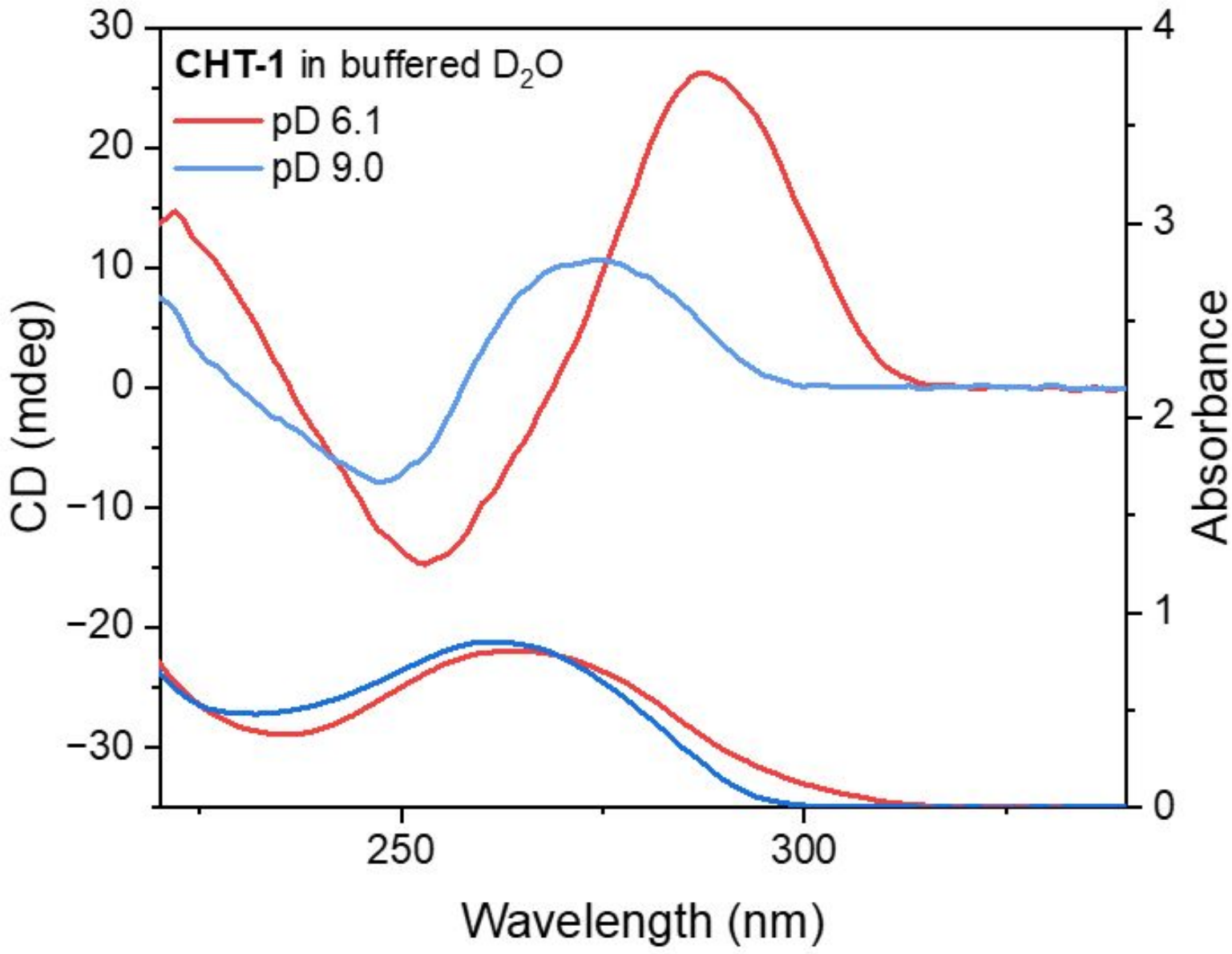

Figure S35. CD (upper data, left axis) and UV-visible absorption (lower data, right axis) spectra of **CHT-1** (3.6 µM) in buffered D_2O at pD 6.1 and 9.0.

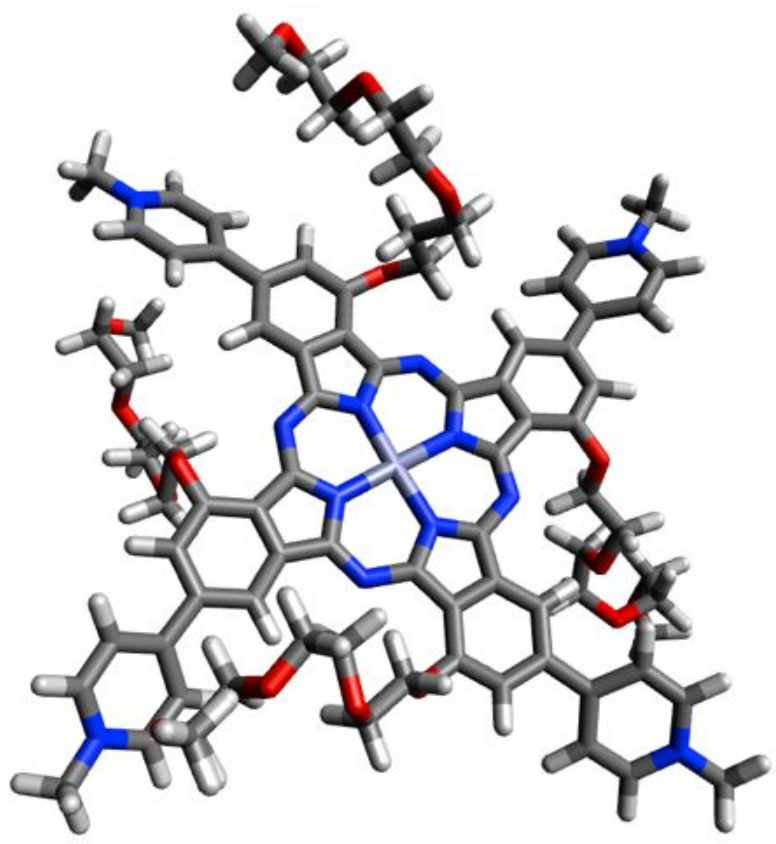

Figure S36: Molecular structure of ZnPc **1** (without counterions) generated using Avogadro 1.2.0.

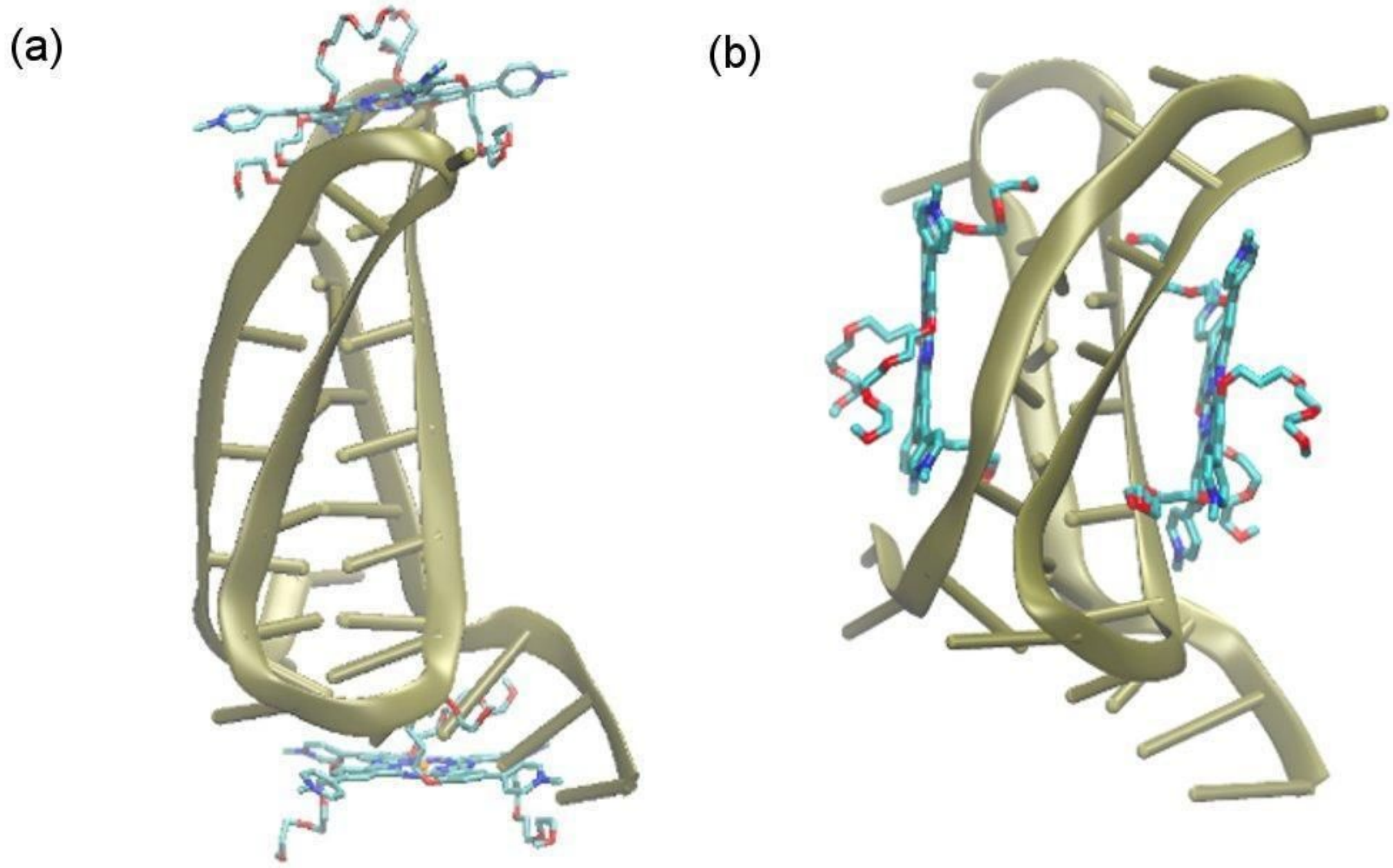

Figure S37: The two structural models considering either end-on (Model 1) or side-on (Model 2) binding of **1** to the iM structure in a 2:1 (**1**:DNA) stoichiometry.

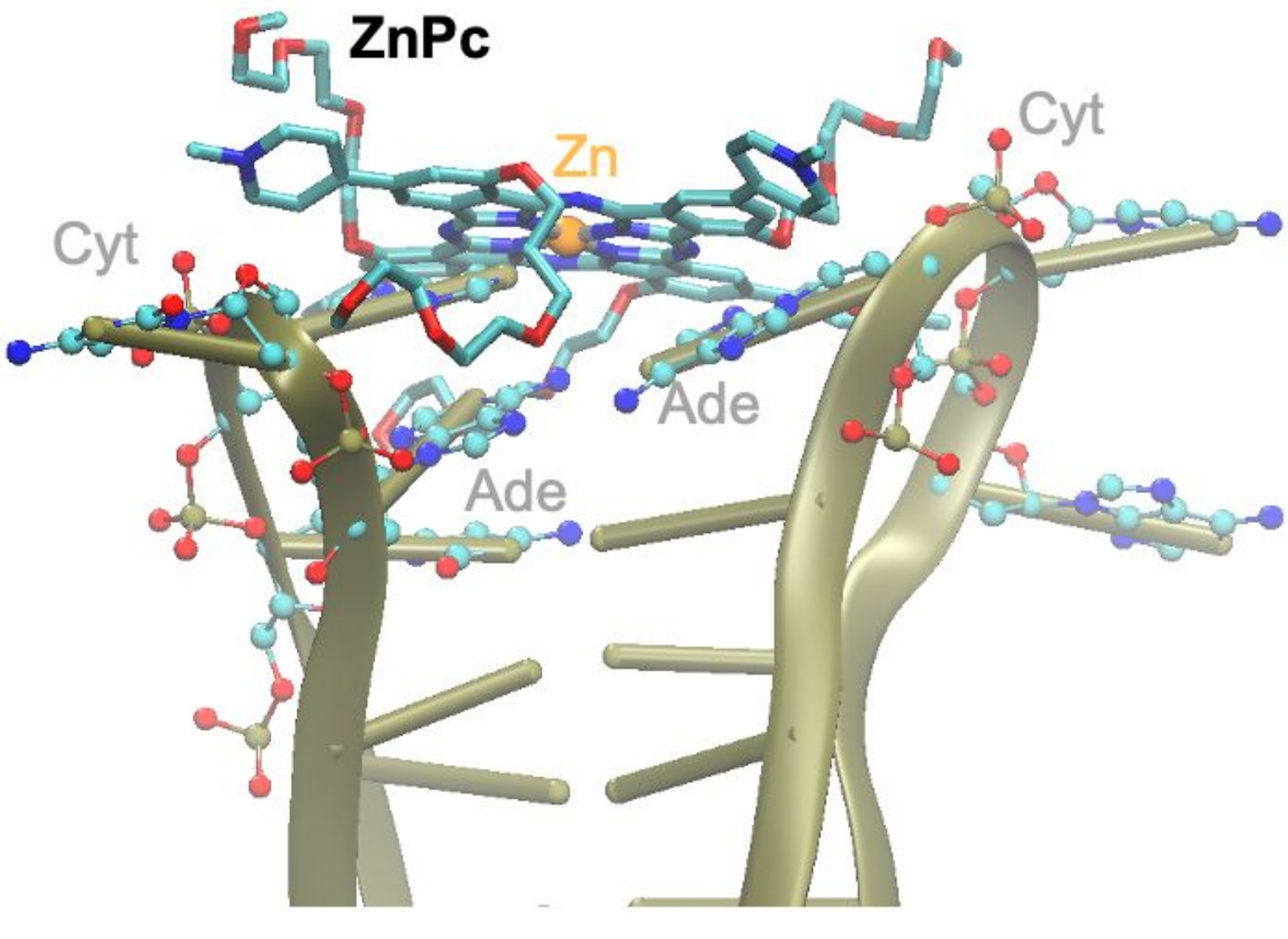

Figure S38: Zoomed in view of end-on (Model 1) of **1** to the iM structure in a 2:1 (**1**:DNA) stoichiometry. Aromatic rings from adenosine (nitrogen shown as dark blue spheres, carbon shown as cyan spheres) stack with the end-on arrangement of ZnPc, but electrostatic interactions between phosphate groups (oxygen shown as red spheres and phosphorus shown as brown spheres) from cytosine and ZnPc are limited, leading to an overall interaction energy between ZnPc and DNA of approx +35 kcal/mol (unfavorable).

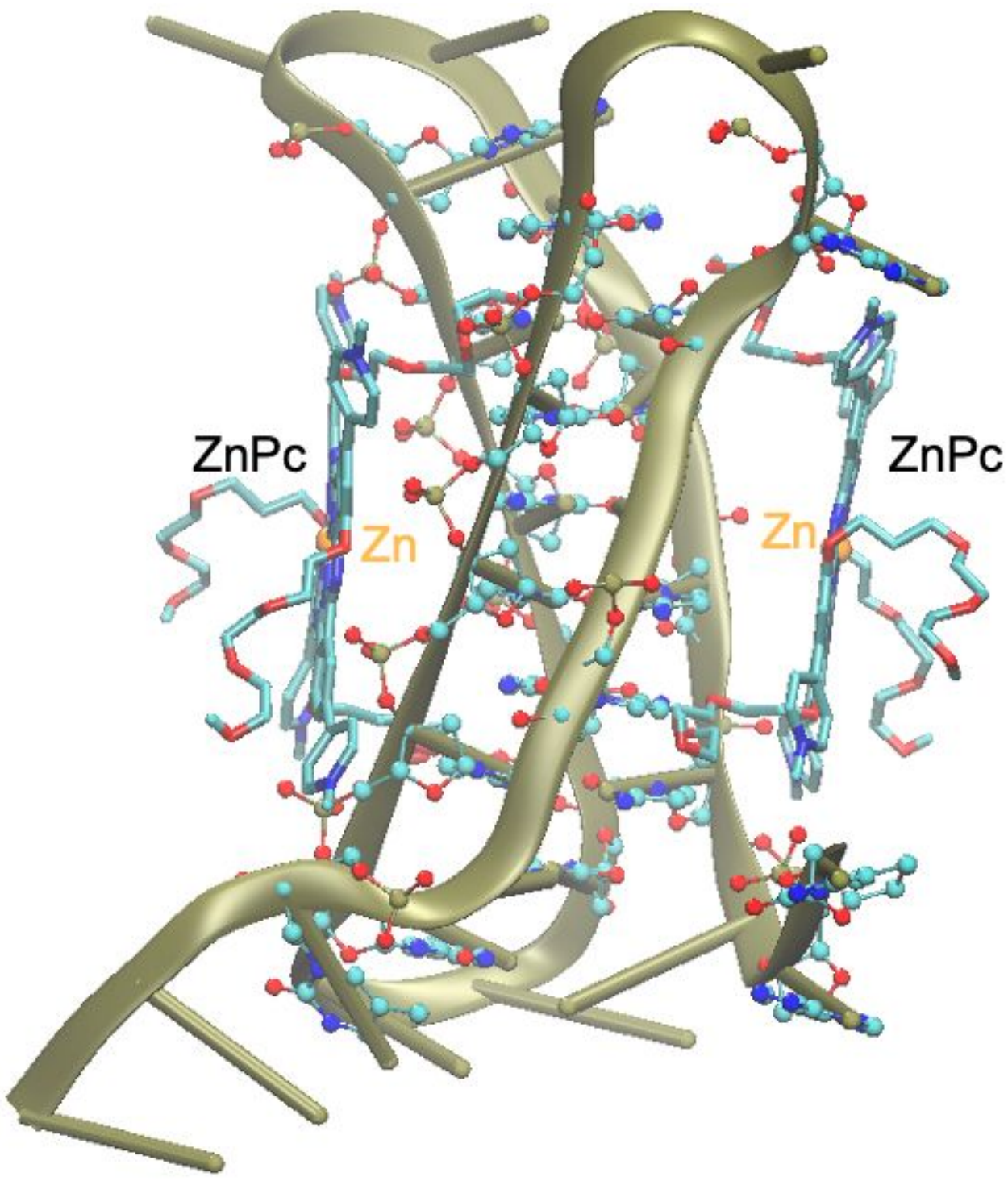

Figure S39: Zoomed in view of side-on (Model 2) binding of **1** to the iM structure in a 2:1 (**1**:DNA) stoichiometry. Side-on arrangement of ZnPc leads to multiple stabilizing electrostatic interactions between positively charged ZnPc and the negatively charged phosphate groups (favorable interaction energy approx -40 kcal/mol).

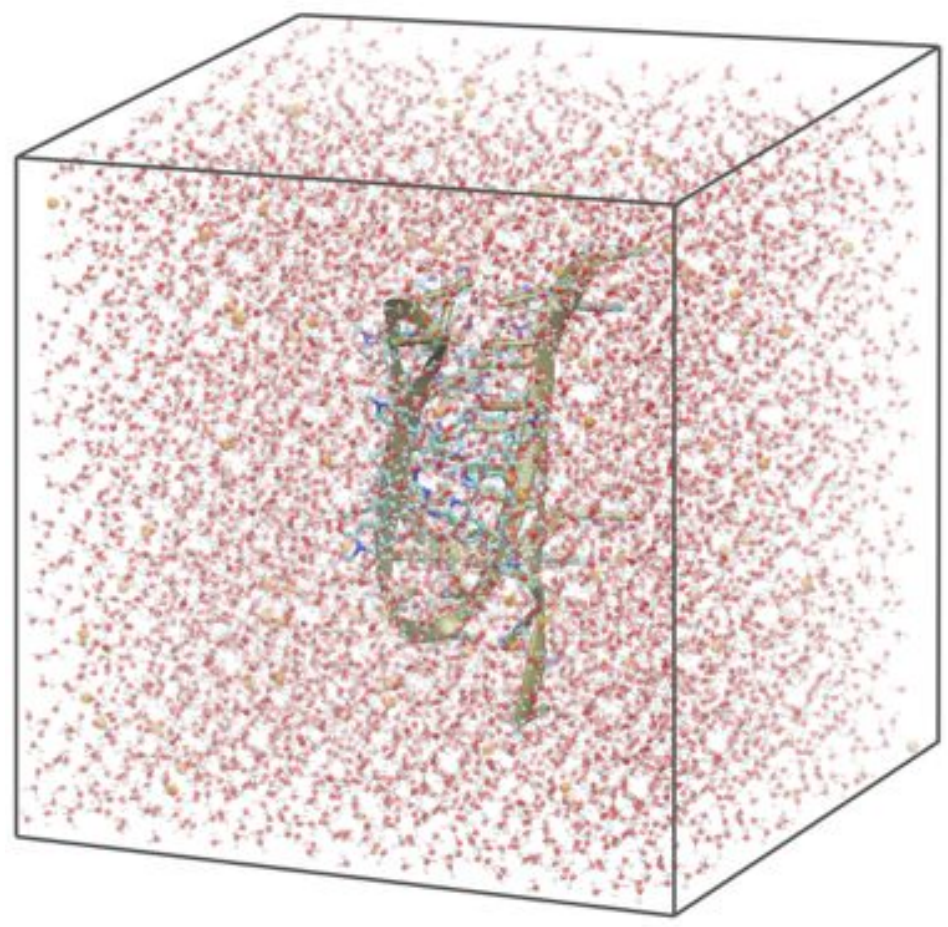

Figure 40: **1**-DNA model solvated in a box of explicit water with 0.05 M K_3PO_4.

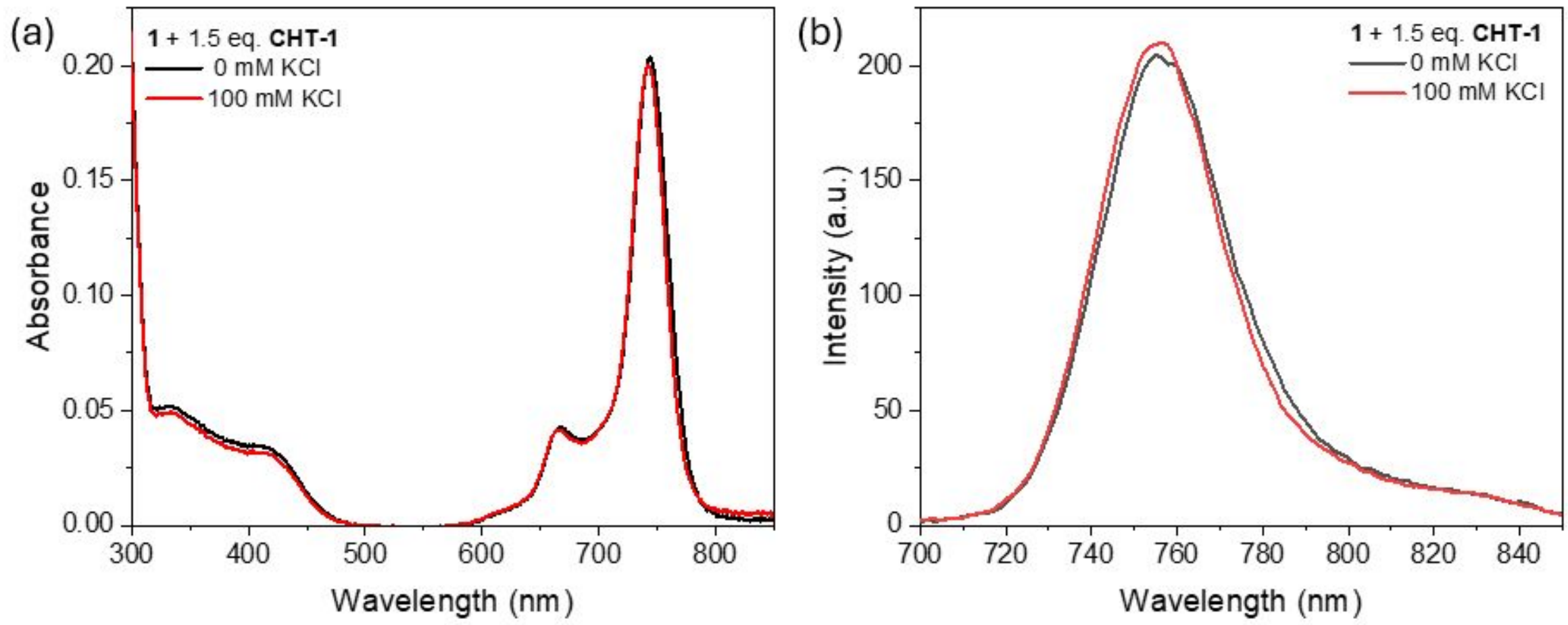

Figure S41: (a) UV-visible absorption and (b) emission spectra (λ_{ex} = 650 nm) of **1** (2.3 μM) and **CHT-2** (3.5 μM) in 50 mM potassium phosphate buffer (pH 5.5) in absence and presence of 100 mM KCl.

S3 Tables

Table S1. Nucleic acid sequences studied

Name	Sequence	Molar Absorptivity at 260 nm per strand (M^{-1} cm^{-1})
**CHT-1**	5′ CCC-TAA-CCC-TAA-CCC-TAA-CCC-TAA 3’	220,400
**CHT-2**	5′ CCC-TAA-CCC-TAA-CCC-TAA-CCC 3’	185,900
**cMYC-1**	5' TTA-CCC-ACC-CTA-CCC-ACC-CTC-A 3'	191,500
**cMYC-2**	5' TTT-TCC-TAC-CCT-TCC-TAC-CCT-AA 3'	198,200
**cMYC-1M**	5' CTC-TCA-CCA-CAC-CAC-ACC-TCT-C 3’	188,200
**C_3T_3**	5' CCC-TTT-CCC-TTT-CCC-TTT-CCC 3'	159,500
**C_3T_5**	5' CCC-TTT-TTC-CCT-TTT-TCC-CTT-TTT-CCC 3'	208,100
**C_3T_7**	5' CCC-TTT-TTT-TCC-CTT-TTT-TTC-CCT-TTT-TTT-CCC 3'	256,700
**C_5T_3**	5’ CCCCC-TTT-CCCCC-TTT-CCCCC-TTT-CCCCC 3’	217,100
**C_{30}**	5’ CCC-CCC-CCC-CCC-CCC-CCC-CCC-CCC-CCC-CCC 3’	216,200
**DDD-AT**	5’ CGC-GAT-ATC-GCG	112,700
**cMYC-G4**	5' TAG-GGA-GGG-TAG-GGA-GGG-T 3'	201,700
**G-HT**	5’ TT-GGG-TTA-GGG-TTA-GGG-TTA-GGG-A 3’	244,300

Table S2. K_{app} and n values from Hill fits of titration between **1** (2.3 μM) and **CHT-1**, **CHT-2**, **cMYC-1**, and **cMYC-1M** (0 – 3.2 μM). n = 2.

DNA	K_{app}	n
CHT-1	617 ± 74 nM	2.5
CHT-2	668 ± 15 nM	2.5
cMYC-1	697 ± 57 nM	2.6
cMYC-1M	407 ± 28 nM	2.5

Table S3. Comparison of absorption and emission spectra of **1** following addition of 1.5 equivalents of the iM-forming sequences in 50 mM potassium phosphate buffer (pH 5.5).

DNA	Abs_Q/Abs_A[a]	λ_Q[b] (nm)	Hypochromism at 330 nm [c]	I/I_0[d]	$\Delta\lambda_{em}$[e]	% Abs 24 h[f]	% Emission 24 h[g]
**CHT-1**	5.6	742	58%	19	7	0	5
**CHT-2**	5.7	742	53%	19	8	0	4
**cMYC-1**	5.5	743	60%	19	10	4	4
**cMYC-2**	4.5	743	68%	13	8	-10	-4
**cMYC-1M**	6.0	743	57%	18	9	10	8
**C_3T_3**	4.0	743	71%	6.1	9	2	10
**C_3T_5**	2.4	741	51%	8.1	9	-12	3
**C_3T_7**	1.8	741	61%	5.3	10	0	15
**C_5T_3**	1.6	740	58%	3.5	9	-9	1
**C_{30}**	7.1	741	51%	27	7	12	13

[a] Ratio of Q-band to aggregate (A) band absorbance. [b] Wavelength of maximum Q-band absorbance following titration. [c] Percentage loss of intensity at 330 nm. [d] Emission intensity at end of titration relative to initial intensity. [e] Shift in maximum emission wavelength from 748 nm in the absence of DNA. [f] Percentage increase in Q-band absorbance 24 h following titration. [g] Percentage increase in emission intensity 24 h following titration

Table S4. Temperature (°C) at 50% of change in absorbance at 260 nm, 295 nm, 687 nm, 742 nm when heated and cooled for CHT-1 with 0.5, 1, and 2 equivalents of **1**. In 20 mM sodium acetate buffer (pH 5.5).

**[iM]:[1]**		**DNA**		**PC**		**Difference**
		**260 nm**	**295 nm**	**687 nm**	**742 nm**	
**1:0.5**	Heating	46.1	45.7	61.1	58.3	
	Cooling	41.1	42.5	58.1	53.7	
	**Average**	**T_M = 43.9 ± 2.4**		**T_A = 57.8 ± 3.1**		**13.9 ± 3.9**
**1:1**	Heating	45.7	45.7	59.7	54.3	
	Cooling	40.9	42.3	55.9	51.5	
	**Average**	**T_M = 43.7 ± 2.4**		**T_A = 55.4 ± 3.4**		**11.7 ± 4.2**
**1:2**	Heating	42.5	52.3	53.7	54.3	
	Cooling	40.3	42.3	49.3	46.1	
	**Average**	**T_M = 44.4 ± 5.4**		**T_A = 50.9 ± 3.9**		**6.5 ± 6.7**

Table S5. Fluorescence lifetimes of **1** (2.3 μM) in the presence of iM-forming sequences (3.5 μM strand) in 50 mM potassium phosphate buffer (pH 5.5) measured via TCSPC.

	τ (ns)	X^2
Buffer (pH 5.5)	1.80	1.07
DMSO	1.93	1.25
**CHT-1**	2.09	1.23
**CHT-2**	2.03	1.27
**cMYC-1**	1.97	1.30
**cMYC-2**	2.02	1.26
**cMYC-1M**	2.01	1.28
**C_3T_5**	2.02	1.24
**C_{30}**	2.06	1.13

Table S6. Photoluminescence quantum yield of **1** in 50 mM potassium phosphate buffer (pH 5.5) or DMSO at r.t.

	**PLQY**
**1** in buffer	0.02%
**1** + 2 eq. cMYC-1 in buffer	5.2%
**1** in DMSO	11.9%

S4 References

(1) Windle, E. R.; Rennie, C. C.; Edkins, R. M.; Quinn, S. J. Role of Secondary Structure and Time-Dependent Binding on Disruption of Phthalocyanine Aggregates by Guanine-Rich Nucleic Acids. *Chem. Eur. J.* **2025**, *31* (2), e202403095. DOI: 10.1002/chem.202403095.

(2) Windle, E. R, MacKay, D. G., Graczyk, D.; Rennie, C. C.; Malakar, P.; Quinn, S. J.; Edkins, R. M. Femto- to Microsecond Excited-State Dynamics of Aggregated and Monomeric Regiopure Zinc and Copper Phthalocyanines. *ChemRxiv* **2026**. DOI: 10.26434/chemrxiv.15005704/v.

(3) Glasoe, P. K.; Long, F. A. Use of Glass Electrodes to Measure Acidities in Deuterium Oxide. *J. Phys. Chem.* **1960**, *64* (1), 188–190. DOI: 10.1021/j100830a521.

(4) Berman, H. M.; Westbrook, J.; Feng, Z.; Gilliland, G.; Bhat, T. N.; Weissig, H.; Shindyalov, I. N.; Bourne, P. E. The Protein Data Bank. *Nucleic Acids Res.* **2000**, *28* (1), 235–242. DOI: 10.1093/nar/28.1.235.

(5) Guneri, D.; Alexandrou, E.; El Omari, K.; Dvořáková, Z.; Chikhale, R. V.; Pike, D. T. S.; Waudby, C. A.; Morris, C. J.; Haider, S.; Parkinson, G. N.; et al. Structural insights into i-motif DNA structures in sequences from the insulin-linked polymorphic region. *Nat. Commun.* **2024**, *15* (1), 7119. DOI: 10.1038/s41467-024-50553-0.

(6) Hanwell, M. D.; Curtis, D. E.; Lonie, D. C.; Vandermeersch, T.; Zurek, E.; Hutchison, G. R. Avogadro: an advanced semantic chemical editor, visualization, and analysis platform. *J. Cheminform.* **2012**, *4* (1), 17. DOI: 10.1186/1758-2946-4-17.

(7) Frisch *et al*. Gaussian 16, Revision A.03. Gaussian, Inc., Wallingford CT (2016).

(8) Grimme, S.; Antony, J.; Ehrlich, S.; Krieg, H. A consistent and accurate ab initio parametrization of density functional dispersion correction (DFT-D) for the 94 elements H-Pu. *J. Chem. Phys.* **2010**, *132* (15). DOI: 10.1063/1.3382344. Grimme, S.; Ehrlich, S.; Goerigk, L. Effect of the damping function in dispersion corrected density functional theory. *J. Comp. Chem.* **2011**, *32* (7), 1456–1465. DOI: 10.1002/jcc.21759.

(9) Vanommeslaeghe, K.; Hatcher, E.; Acharya, C.; Kundu, S.; Zhong, S.; Shim, J.; Darian, E.; Guvench, O.; Lopes, P.; Vorobyov, I.; et al. CHARMM general force field: A force field for drug-like molecules compatible with the CHARMM all-atom additive biological force fields. *J. Comp. Chem.* **2010**, *31* (4), 671–690. DOI: 10.1002/jcc.21367.

(10) Humphrey, W.; Dalke, A.; Schulten, K. VMD: Visual molecular dynamics. *Journal of Molecular Graphics* **1996**, *14* (1), 33–38. DOI: 10.1016/0263-7855(96)00018-5.

(11) Jo, S.; Kim, T.; Iyer, V. G.; Im, W. CHARMM-GUI: A web-based graphical user interface for CHARMM. *J. Comp. Chem.* **2008**, *29* (11), 1859–1865. DOI: 10.1002/jcc.20945.

(12) MacKerell, A. D., Jr.; Bashford, D.; Bellott, M.; Dunbrack, R. L., Jr.; Evanseck, J. D.; Field, M. J.; Fischer, S.; Gao, J.; Guo, H.; Ha, S.; et al. All-Atom Empirical Potential for Molecular Modeling and Dynamics Studies of Proteins. *J. Phys. Chem. B.* **1998**, *102* (18), 3586–3616. DOI: 10.1021/jp973084f.

(13) Brooks, B. R.; Bruccoleri, R. E.; Olafson, B. D.; States, D. J.; Swaminathan, S.; Karplus, M. CHARMM: A program for macromolecular energy, minimization, and dynamics calculations.

J. Comp. Chem. **1983**, *4* (2), 187–217. DOI: 10.1002/jcc.540040211.

(14) Jorgensen, W. L.; Chandrasekhar, J.; Madura, J. D.; Impey, R. W.; Klein, M. L. Comparison of simple potential functions for simulating liquid water. *J. Chem. Phys.* **1983**, *79* (2), 926–935. DOI: 10.1063/1.445869.

(15) Liu, L.; Kim, B. G.; Feroze, U.; Macgregor, R. B., Jr.; Chalikian, T. V. Probing the Ionic Atmosphere and Hydration of the c-MYC i-Motif. *J. Am. Chem. Soc.* **2018**, *140* (6), 2229–2238. DOI: 10.1021/jacs.7b11537.

(16) Gao, B.; Hou, X.-M. Opposite Effects of Potassium Ions on the Thermal Stability of i-Motif DNA in Different Buffer Systems. *ACS Omega* **2021**, *6* (13), 8976–8985. DOI: 10.1021/acsomega.0c06350.
